# Supplementary material for: Visceral Adipose Tissue Immune Homeostasis Is Regulated by the Crosstalk between Adipocytes and Dendritic Cell Subsets
Source: Cell Metab. 2018 Mar 6;27(3):588–601.e4. doi: 10.1016/j.cmet.2018.02.007 (PMC5846800; doi:10.1016/j.cmet.2018.02.007)
Supplement: Document S1. Figures S1–S6 and Table S1 [file mmc1.pdf]

Cell Metabolism, Volume *27*

Supplemental Information

Visceral Adipose Tissue Immune Homeostasis Is Regulated by the Crosstalk between Adipocytes and Dendritic Cell Subsets

Claire E. Macdougall, Elizabeth G. Wood, Jakob Loschko, Valeria Scagliotti, Féaron C. Cassidy, Mark E. Robinson, Niklas Feldhahn, Leandro Castellano, Mathieu-Benoit Voisin, Federica Marelli-Berg, Carles Gaston-Massuet, Marika Charalambous, and M. Paula Longhi

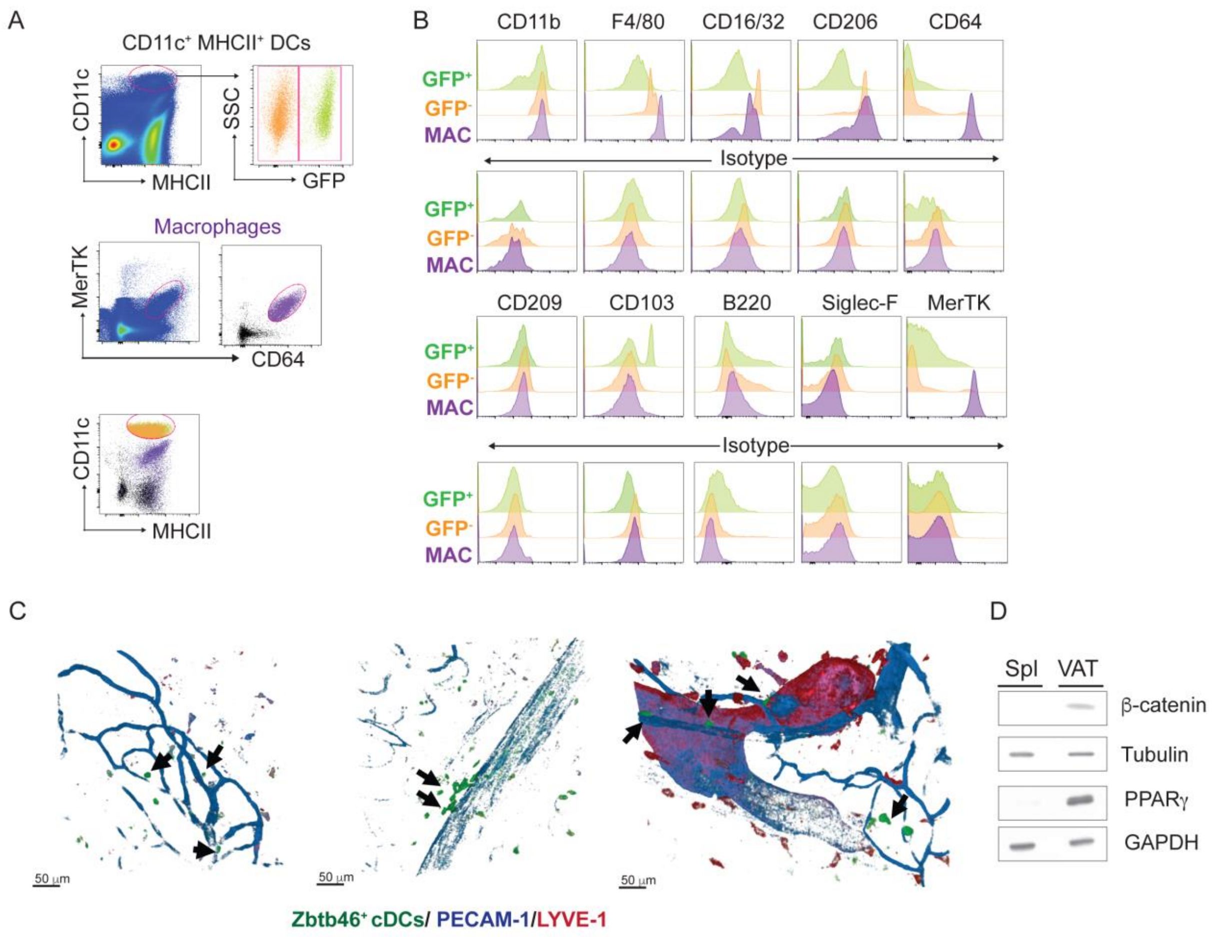

Supplementary Figure 1. Phenotypic characterization of cDCs in VAT. Related to Figure 1. VAT was isolated from Zbtb46-GFP$^+$ mice. (A) cDCs were identified as MHCII$^+$ CD11c$^+$ GFP$^+$ cells and macrophages as MerTK$^+$ CD64$^+$ cells. (B) Phenotypic characterization of MHCII$^+$ CD11c$^+$ GFP$^+$, MHCII$^+$ CD11c$^+$ GFP$^-$ cells and macrophages. Dot plots and histograms are representative of 3 independent experiments. (C) Mesenteric VAT and vessels from Zbtb46-GFP$^+$ mice were imaged by *ex vivo* confocal microscopy. Tissue was visualized viewed using a Leica SP8 confocal microscope. GFP$^+$ cDCs (green) located in close proximity (black arrows) to PECAM-1$^+$ capillary vessels (BV, blue) of the VAT (left picture) but also close to mesenteric afferent lymphatics (LV, middle picture) and

LYVE-1^{+} initial lymphatic vessels (iLV, red) near the gut (right picture). (D) Expression of active β-catenin and PPARγ in spleen and VAT detected by Western blot representative of 3 independent experiments.

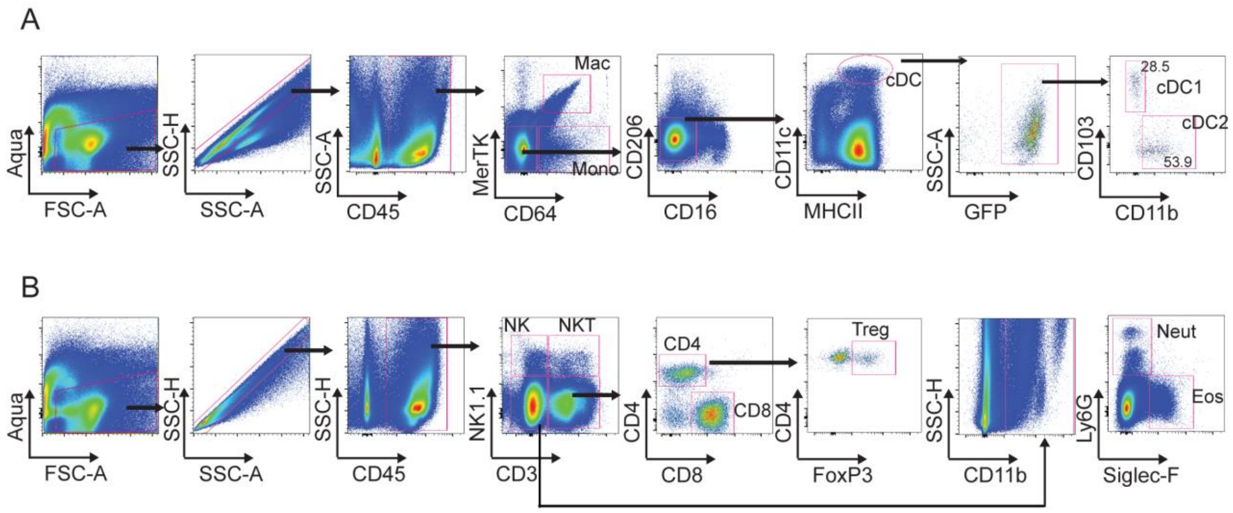

Supplementary Figure 2. Gating strategy. Related to Figure 3 and 5. VAT was isolated from Zbtb46-GFP$^+$ mice and immune populations were analyzed by flow cytometry. Dot plots show the gating strategy to identify cDCs, monocytes and macrophages (A, stain 1) as well as T cell populations, neutrophils, eosinophils, NK cells, NK T cells (B, stain 2).

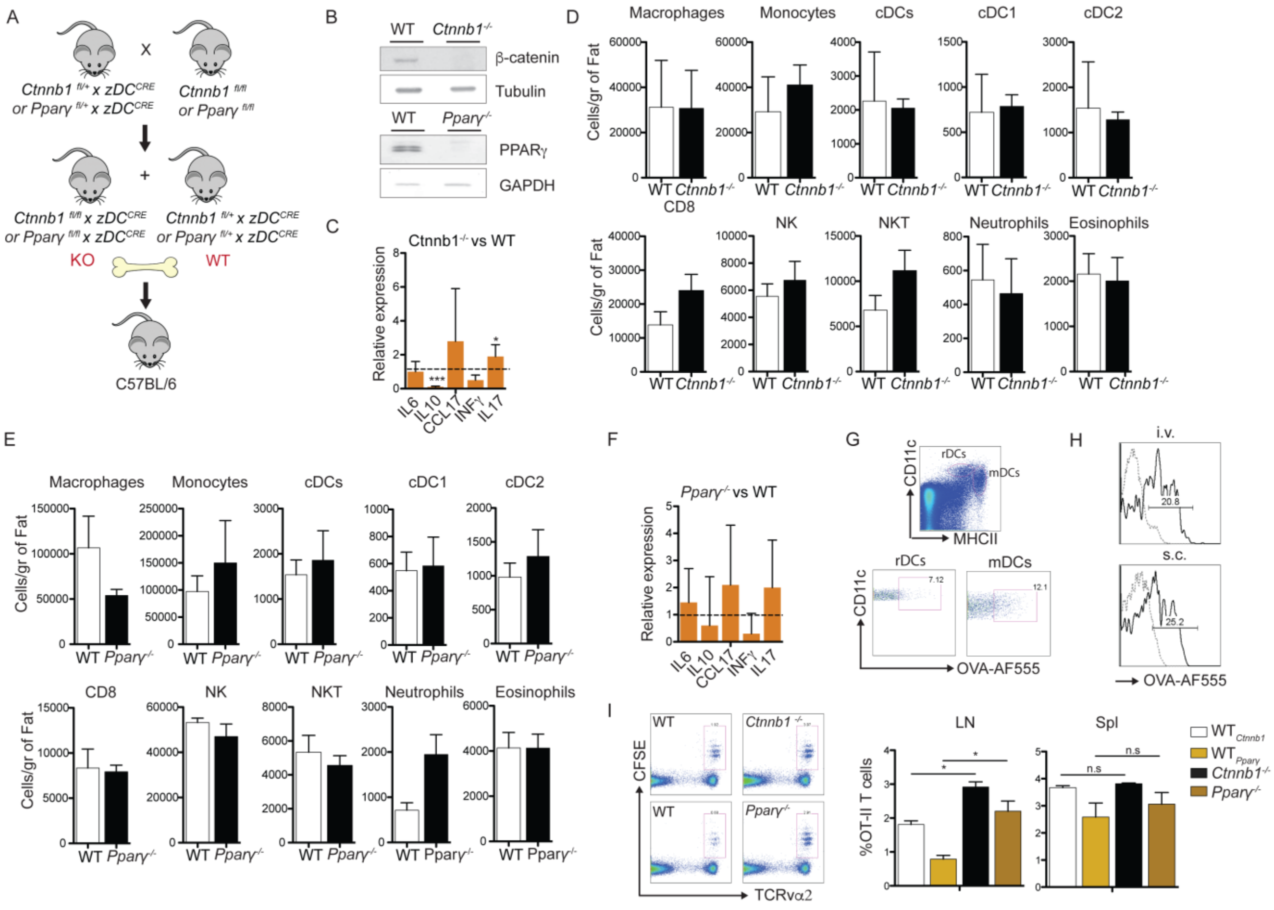

Supplementary Figure 3. Characterization of VAT-immune infiltrates in *Ctnnb1*$^{-/-}$ and *Pparγ*$^{-/-}$ mice on chow diet. Related to Figure 3. (A) Scheme showing experimental mice model**.** (B) Western blot showing lack of β-catenin and PPARγ expression in *Ctnnb1*$^{-/-}$ and *Pparγ*$^{-/-}$ mice respectively. (C) Quantitative RT PCR analysis for the gene expression in SVF from *Ctnnb1*$^{-/-}$ and WT mice in resting conditions. Expression levels of all genes were normalized against GAPDH RNA. Bars represent mRNA expression of conditional KO compared to WT mice, which was set at 1 as indicated with a dotted line. Error bars indicate the geometric mean of 5 biological replicates. (D-E) VAT was digested from lean *Ctnnb1*$^{-/-}$ (D), *Pparγ*$^{-/-}$ (E) and respective WT control mice. Total numbers of infiltrated immune cells identified as described in Supplementary Figure 2, were quantified by flow cytometry. Bars represent the mean ± SEM (n=6). (F) As in (C) but quantitative RT PCR

analysis for the gene expression in SVF from *Pparγ*$^{-/-}$ and WT mice in resting conditions. (G) Dot plots indicate *in vivo* AF555-OVA uptake by resident and migratory cDCs in LN. (H) Histograms indicate *in vivo* AF555-OVA uptake by VAT-cDCs after i.v. and s.c. immunization. (I) CFSE-labeled OT-II cells were transferred i.v. one day before inoculation of 200 μg OVA plus 1 μg of LPS i.p. Two days later, OT-II cell proliferation was assessed by flow cytometry. Dot plots show CFSE dilution of OT-II cells in LN (left). Bar graphs represent the average frequency of division of proliferating OT-II T cells in LN and spleen from 3 independent experiments (n=6).

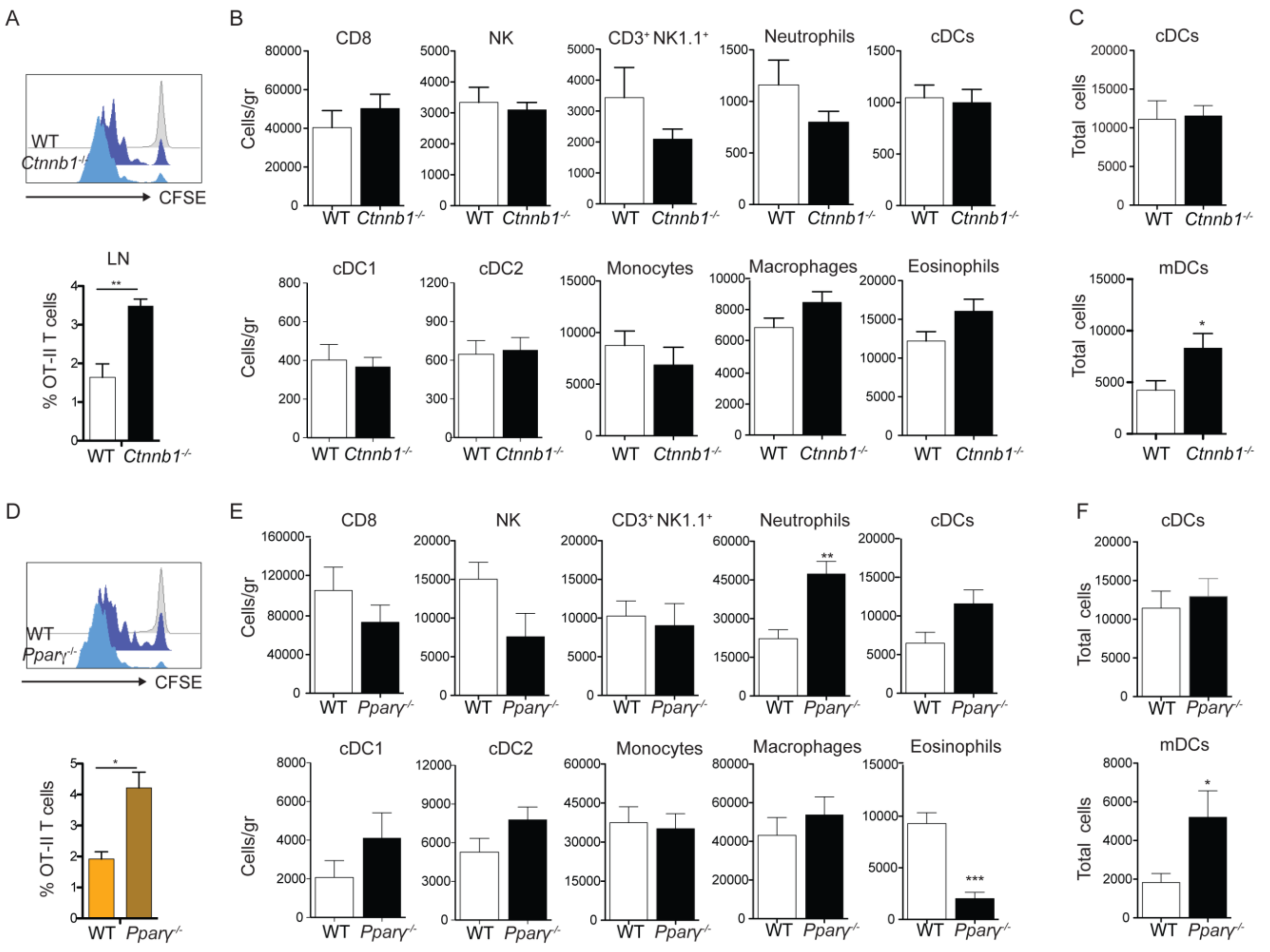

Supplementary Figure 4. Characterization of VAT-immune infiltrates in *Ctnnb1*$^{-/-}$ and *Pparγ*$^{-/-}$ mice on WD. Related to Figure 5. (A) CFSE-labeled OT-II cells were transferred i.v. one day before inoculation of 200 µg OVA i.p in *Ctnnb1*$^{-/-}$ and WT control mice. Three days later, OT-II cell proliferation was assessed by flow cytometry. Histogram shows CFSE dilution of OT-II cells in LN. Bar graphs represent the average frequency of division of proliferating OT-II T cells in LN in 2 independent experiments. *Ctnnb1*$^{-/-}$ (B-C), *Pparγ*$^{-/-}$ (E-F) and respective control mice were fed a WD for 12 weeks. Immune cells were identified as described in Supplementary Figure 2. Bar graphs represent the total numbers of immune cells per gram of VAT (B) and total cell numbers in LN (C) from *Ctnnb1*$^{-/-}$ and WT control mice. (D) *In vivo* OVA presentation as in (A) but in *Pparγ*$^{-/-}$ and WT control mice. (E-

F) Similar to (B-C) but immune cell numbers were quantified in *Pparγ*$^{-/-}$ and WT control mice. Bars represent the mean ± SEM (n=6).

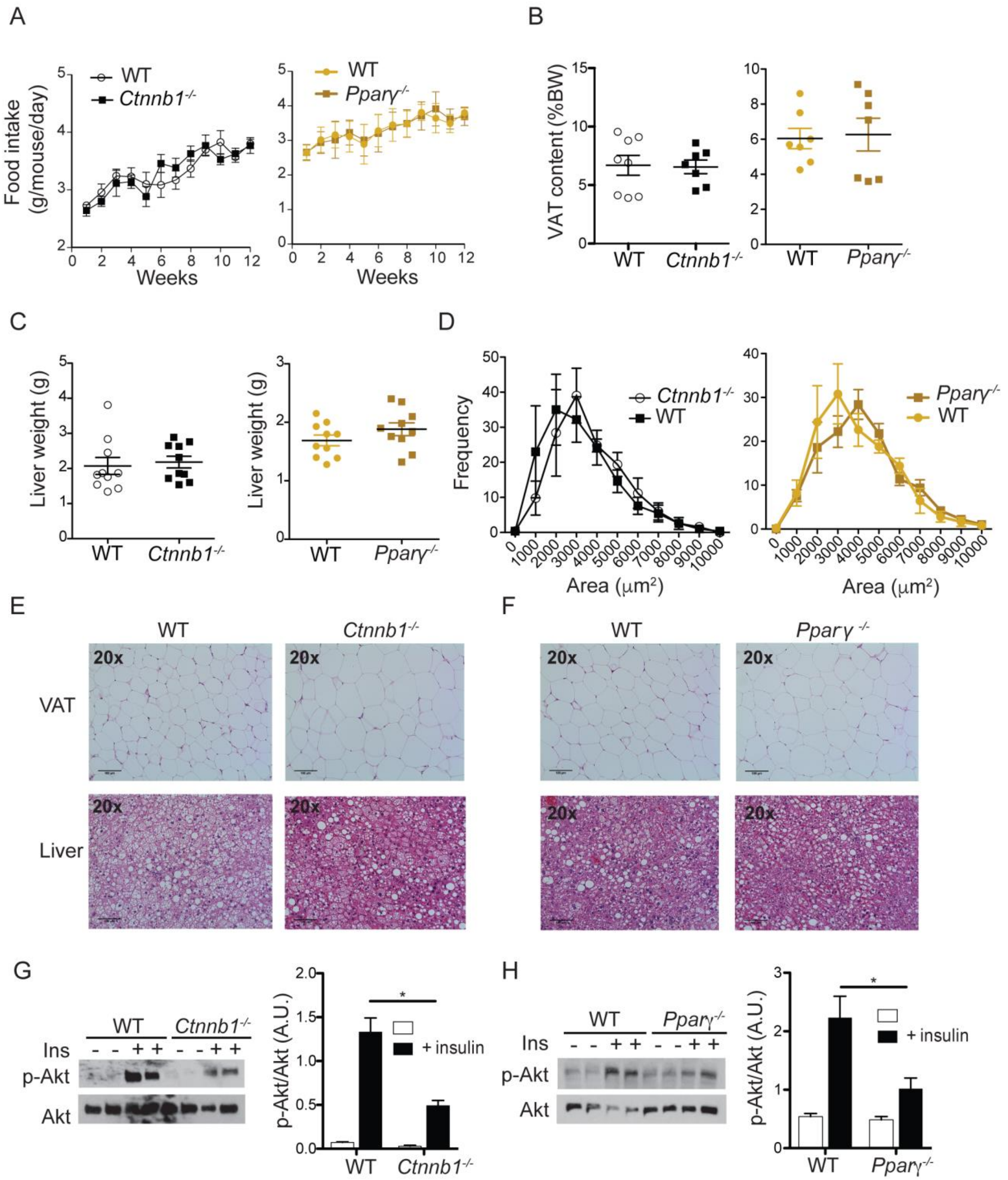

Supplementary Figure 5. Minor changes in adipocyte function and size in *Ctnnb1*$^{-/-}$ and *Pparγ*$^{-/-}$ mice on WD. Related to Figure 6. *Ctnnb1*$^{-/-}$ and *Pparγ*$^{-/-}$ and WT control

mice were fed a WD for 12 weeks. (A) Food intake was monitored weekly. Bars represent the mean ± SEM (n=10). (B) VAT content was calculated as percentage of body weight. Graphs represent the mean ± SEM. (C) Liver weight at 12 weeks of WD. Each dot represents an individual mouse. (D) Frequency distribution of adipocyte area isolated from VAT. Frequency of cell area is expressed in number of cells per area bin using ImageJ Adiposoft software. (E-F) Representative H&E staining from VAT and liver from *Ctnnb1*$^{-/-}$ (E) and *Pparγ*$^{-/-}$ (F) and WT control mice. (G-H) Western blot analysis of Liver extracts showing phosphor-(Ser473) Akt (p-Akt) levels under control and insulin treatment. Graphs show densitometry of p-Akt/Akt ratios in arbitrary units (A.U.).*p<0,05, **p<0,01 and ***p<0.0005.

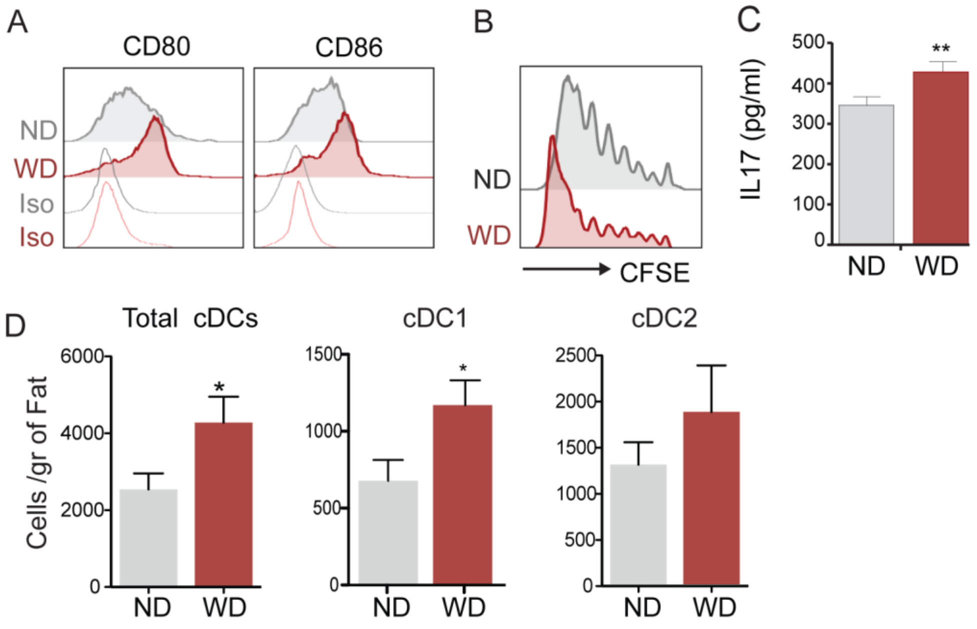

Supplementary Figure 6. WD induces activation of VAT-cDCs. Related to Figure 7.

Zbtb46-GFP$^+$ mice were fed a chow or WD diet for 12 weeks. (A) Expression of CD80 and CD86 in VAT-cDC isolated from chow or WD fed mice. Histograms are representative of 3 independent experiments. (B) VAT-cDCs were purified and plated with purified allogeneic Balb/c CD4$^+$ T cells (1:5) for 5 days. T cell proliferation was assessed by CFSE dilution by flow cytometry. Histograms are representative of 3 independent experiments. (C) IL-17 production by allogeneic T cells was measured in supernatant by ELISA. (D) Numbers of VAT-cDC were calculated by flow cytometry. Bars represent the mean ± SEM (n=10).

Table S1.
Related to STAR Methods Key Resource Table (Oligonucleotides section): List of primers used in this paper

Oligonucleotide name	Sequence (5' to 3')
IL10_forward	AAA CAA AGG ACC AGC TGG AC
IL10_reverse	TTC CGA TAA GGC TTG GCA AC
TNFα_forward	TCG TAG CAA ACC ACC AAG TG
TNFα_reverse	TTT GAG ATC CAT GCC GTT GG
IL6_forward	TAG TCC TTC CTA CCC CAA TTT CC
IL6_reverse	TTG GTC CTT AGC CAC TCC TTC
IL1_forward	AAC CTG CTG GTG TGT GAC GTT C
IL1_reverse	CAG CAC GAG GCT TTT TTG TTG T
INFγ_forward	CAG CAA CAG CAA GGC GAA A
INFγ_reverse	CTG GAC CTG TGG GTT GTT GAC
IL17_forward	AAA GCT CAG CGT GTC CAA AC
IL17_reverse	TTC TGG AGC TCA CTT TTG CG
CCL2_forward	GCT GGA GCA TCC ACG TGT T
CCL2_reverse	ATC TTG CTG GTG AAT GAG TAG CA
CCL17_forward	GGA TGC CAT CGT GTT TCT GA
CCL17_reverse	GCC TTC TTC ACA TGT TTG TCT TTG
Apidoq_forward	GAC GTT ACT ACA ACT GAA GAG C
Apidoq_reverse	CAT TCT TTT CCT GAT ACT GGT C
Leptin_forward	TGA GTT TGT CCA AGA TGG ACC
Leptin_reverse	GCC ATC CAG GCT CTC TGG
GAPDH_forward	GGC TCA TGA CCA CAG TCC A
GAPDH_reverse	CAC ATT GGG GGT AGG AAC AC
TGFβ_forward	CCCGAAGCGGACTACTATGC
TGFβ_reverse	ATAGATGGCGTTGTTGCGGT
WNT1_ forward	ATT TTG CGC TGT GAC CTC TT
WNT1_reverse	AGC AAC CTC CTT TCC CAC TT
WNT2_forward	GGT CAG CTC TTC ATG GTG GT
WNT2_reverse	GGA ACT GGT GTT GGC ACT CT
WNT3a_forward	TCG GAG ATG GTG GTA GAG AAA
WNT3a_reverse	CGC AGA AGT TGG GTG AGG
WNT5b_forward	TCT CCG CCT CAC AAA AGT CT
WNT5b_reverse	CAC AGA CAC TCT CAA GCC CA
WNT6_forward	GCG GAG ACG ATG TGG ACT TC
WNT6_reverse	ATG CAC GGA TAT CTC CAC GC
WNT7a_forward	GAC AAA TAC AAC GAG GCC GT
WNT7a_reverse	GGC TGT CTT ATT GCA GGC TC
WNT8b_forward	CCA GAG TCC CGG GAG GTA G
WNT8b_reverse	GAG ATG GAG CGG AAG GTG T
WNT9a_forward	GGA CAA CCT CAA GTA CAG CAG
WNT9a_reverse	TCC ACT CCA GCC TTT ATC ACC
WNT9b_forward	ACA GCA CCA AGT TCC TCA GC
WNT9b_reverse	CTT GCA GGT TGT TCT CAG GC
WNT10a_forward	CCA CTC CGA CCT GGT CTA CTT TG

WNT10a_reverse	TGC TGC TCT TAT TGC ACA GGC
WNT10b_forward	AAT GCG GAT CCA CAA CAA CA
WNT10b_reverse	TTC CAT GGC ATT TGC ACT TC
WNT11_forward	TGC TTG ACC TGG AGA GAG GT
WNT11_reverse	AGC CCG TAG CTG AGG TTG T
Fabp4_forward	ATGTGCGACCAGTTTGTG
Fabp4_reverse	TTTGCCATCCCACTTCTG
Fabp5_forward	TGAAGGAGCTAGGAGTGGGAA
Fabp5_reverse	TGCACCATCTGTAAAGTTGCAG
Dgat1_forward	GGCCTTCTTCCACGAGTACC
Dgat1_reverse	GGCCTCATAGTTGAGCACG
CD36_forward	TCTTTCCTGCAGCCCAATG
CD36_reverse	AGCCTCTGTTCCAACTGATAGTGA
