# Supplementary material for: Visceral Adipose Tissue Immune Homeostasis Is Regulated by the Crosstalk between Adipocytes and Dendritic Cell Subsets
Source: Cell Metab. 2018 Mar 6;27(3):588–601.e4. doi: 10.1016/j.cmet.2018.02.007 (PMC5846800; doi:10.1016/j.cmet.2018.02.007)
Supplement: Document S2. Article plus Supplemental Information [file mmc2.pdf]

Cell Metabolism

Article

# Visceral Adipose Tissue Immune Homeostasis Is Regulated by the Crosstalk between Adipocytes and Dendritic Cell Subsets

## Graphical Abstract

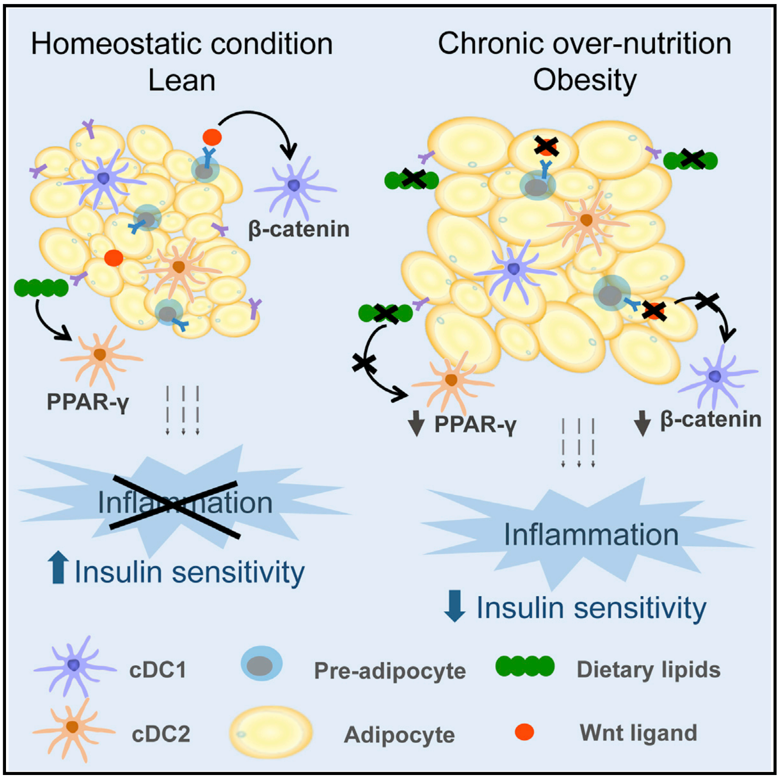


## Authors

Claire E. Macdougall, Elizabeth G. Wood, Jakob Loschko, ..., Carles Gaston-Massuet, Marika Charalambous, M. Paula Longhi

## Correspondence

m.longhi@qmul.ac.uk

## In Brief

Macdougall et al. report key mechanisms that control the immune function of conventional dendritic cells in visceral adipose tissue. The upregulation of adipocyte-related pathways in conventional dendritic cells promotes an anti-inflammatory phenotype in visceral adipose tissue under homeostatic conditions and delays the onset of obesity-induced inflammation and insulin resistance.

## Highlights

- VAT-cDCs acquire a tolerogenic phenotype by upregulating adipocyte-related pathways
- Activation of β-catenin and PPARγ in cDC subsets promotes anti-inflammatory VAT
- Combined, they delay the onset of obesity-induced inflammation and insulin resistance
- Chronic over-nutrition curtails β-catenin and PPARγ pathways, fueling cDC activation

## Data Resources

GSE37448

Macdougall et al., 2018, Cell Metabolism *27*, 588–601
March 6, 2018 © 2018 The Author(s). Published by Elsevier Inc.
https://doi.org/10.1016/j.cmet.2018.02.007

Cell Metabolism
Article

# Visceral Adipose Tissue Immune Homeostasis Is Regulated by the Crosstalk between Adipocytes and Dendritic Cell Subsets

Claire E. Macdougall,[1,5] Elizabeth G. Wood,[1,5] Jakob Loschko,[2] Valeria Scagliotti,[1] Féaron C. Cassidy,[1] Mark E. Robinson,[3,4] Niklas Feldhahn,[3] Leandro Castellano,[4] Mathieu-Benoit Voisin,[1] Federica Marelli-Berg,[1] Carles Gaston-Massuet,[1] Marika Charalambous,[1] and M. Paula Longhi[1,6,*]
[1]William Harvey Research Institute, Barts, and the London School of Medicine and Dentistry, Queen Mary University of London, London EC1M 6BQ, UK
[2]Laboratory of Molecular Immunology, The Rockefeller University, New York, NY 10065, USA
[3]Centre for Haematology, Department of Medicine, Imperial College London, W12 0NN London, UK
[4]Department of Surgery and Cancer, Imperial College London, Imperial Centre for Translational and Experimental Medicine (ICTEM), Hammersmith Hospital, London W12 0NN, UK
[5]These authors contributed equally
[6]Lead Contact
*Correspondence: m.longhi@qmul.ac.uk
https://doi.org/10.1016/j.cmet.2018.02.007

## SUMMARY

**Visceral adipose tissue (VAT) has multiple roles in orchestrating whole-body energy homeostasis. In addition, VAT is now considered an immune site harboring an array of innate and adaptive immune cells with a direct role in immune surveillance and host defense. We report that conventional dendritic cells (cDCs) in VAT acquire a tolerogenic phenotype through upregulation of pathways involved in adipocyte differentiation. While activation of the Wnt/β-catenin pathway in cDC1 DCs induces IL-10 production, upregulation of the PPARγ pathway in cDC2 DCs directly suppresses their activation. Combined, they promote an anti-inflammatory milieu *in vivo* delaying the onset of obesity-induced chronic inflammation and insulin resistance. Under long-term over-nutrition, changes in adipocyte biology curtail β-catenin and PPARγ activation, contributing to VAT inflammation.**

## INTRODUCTION

Visceral adipose tissue (VAT) is a highly active metabolic and endocrine organ that secretes cytokines and bioactive mediators such as adiponectin, which influences not only body weight homeostasis, but also insulin sensitivity, inflammation, and lipid metabolism. VAT can respond rapidly and dynamically to alterations in nutrient intake through adipocyte expansion (hypertrophy) and adipogenesis (hyperplasia) buffering most of the excess or deficit of energy. In mice, during the early stage of high-fat diet (HFD) exposure, VAT expands at the expense of adipocyte hypertrophy and it is not until after 8 weeks that adipocyte hyperplasia can be observed (Wang et al., 2013b). The expansion and renewal of adipocytes are tightly regulated at the transcriptional level. In mature adipocytes, peroxisome proliferator-activated receptor-γ (PPARγ) regulates lipid accumulation during hypertrophy (Wang et al., 2013a), while the Wnt/β-catenin pathway controls adipocyte hyperplasia (Christodoulides et al., 2009).

VAT contains immune cells that work in cooperation with adipocytes to maintain the overall metabolism and physiology of the organ. Under homeostatic conditions, resident immune cells such as macrophages and regulatory T cells (Tregs) produce anti-inflammatory cytokines that limit inflammation and maintain glucose homeostasis (Cipolletta et al., 2012). In addition, macrophages are present in high numbers in VAT and fulfill housekeeping functions by removing excess lipids and dead adipocytes (Chawla et al., 2011). Under chronic over-nutrition, VAT expansion creates an environmental milieu that promotes low-grade inflammation with local production of IL-6, TNFa, and IL-1, and the influx of pro-inflammatory cells (Schipper et al., 2012). The immune regulatory network in VAT is disrupted with a decrease in numbers of Tregs and eosinophils and increased recruitment of activated T cells, IFNγ-producing natural killer (NK) cells, and inflammatory macrophages (Schipper et al., 2012). This meta-inflammation has been implicated as an important etiological factor in the development of insulin resistance and vascular complications.

The presence of conventional dendritic cells (cDCs) in VAT has been suggested in numerous studies, but their role in VAT inflammation and metabolic homeostasis remains elusive. cDCs are usually defined as $CD11c^{hi}$ $MHCII^{+}$-expressing cells. However, the surface marker CD11c has been used to identify inflammatory VAT-infiltrated macrophages (Patsouris et al., 2008). This phenotypic overlap has made it difficult to accurately characterize and study cDCs in VAT. Under prolonged over-nutrition, $MHCII^{+}CD11c^{+}$ cells have been shown to process and present antigens to T cells and to induce Th17 responses. Depletion of all $CD11c^{+}$ cells (including $CD11c^{+}$ monocytes/macrophages) resulted in a rapid normalization of insulin sensitivity and decrease of pro-inflammatory cytokines in obese mice (Bertola et al., 2012; Patsouris et al., 2008; Stefanovic-Racic et al.,


This is an open access article under the CC BY license (http://creativecommons.org/licenses/by/4.0/).

2012), suggesting an inflammatory phenotype. Less is known about their role in homeostatic conditions. Early work suggested a preferential recruitment of dendritic cells to perinodal adipose tissue (Mattacks et al., 2004). More recently, elegant work from Randolph and colleagues demonstrated that cDCs resident in perinodal adipose tissue continuously sample lymph content, including soluble antigens traveling from the tissue to the draining lymph node (dLN) and hence contributing to the immune surveillance of neighboring tissues (Kuan et al., 2015). However, the immune phenotype and the underlying molecular mechanisms that control the immune function of VAT-cDCs in resting conditions have not yet been explored.

Here we report that steady-state cDCs acquire an anti-inflammatory phenotype in VAT, which is regulated by similar pathways that control adipocyte expansion. Activation of the Wnt/β-catenin pathway in cDC1 DC subset induces the production of IL-10, while expression of PPARγ in cDC2 subset suppresses the onset of local inflammatory responses. Deletion of β-catenin and PPARγ in cDCs increases VAT-cDC maturation and increases T cell recruitment in lean and obese conditions. The absence of these regulatory pathways accelerates obesity-induced chronic inflammation and insulin resistance. Interestingly, under persistent over-nutrition, adipocyte hyperplasia decreases the availability of β-catenin ligands for cDC1, while expression of PPARγ in cDC2 is reduced, suggesting a possible mechanism for the pro-inflammatory switch of VAT-cDCs in obesity.

## RESULTS

### cDC1 and cDC2 DC Subsets Differentially Upregulate the β-Catenin and PPARγ Pathways in VAT

The promiscuous expression of the markers MHCII and CD11c within phagocytic cell populations in tissues has hampered the study of cDCs in VAT. To understand the distinct contribution of dendritic cells *in vivo*, new reporter mice have recently been generated that are based on the expression of the highly cDC-specific Zbtb46 promoter (Zbtb46-GFP and Zbtb46-Cre) (Loschko et al., 2016). By using the Zbtb46-GFP mice, which is expressed on cDCs and the MHCII$^{-}$ pre-cDCs, we observed that, unlike in the spleen, approximately 40% of all CD11c$^{hi}$ MHCII$^{+}$ cells are cDCs (Figures 1A and 1B). The remaining cells are characterized by high expression of CD16/32 and CD11b and negative expression of MerTK and CD64 (Figures S1A and S1B). CD16/32 can be expressed by monocytes and monocyte-derived DCs (mo-DCs) (Menezes et al., 2016). However, they are negative for the mo-DC marker CD206 (Figure S1B), suggesting that they may be monocytes with low CD64 expression. Further future characterization is required, which is beyond the scope of this paper. In concordance with previous reports, Zbtb46-GFP$^{+}$ cDCs are strategically located close to blood and lymphatic vessels, consistent with their proposed role in antigen sampling (Figure S1C) (Kuan et al., 2015).

cDCs are present in two main subsets, cDC1 (CD103$^{+}$) and cDC2 (CD11b$^{+}$), which have been shown to have both distinct and overlapping functions (Merad et al., 2013). We asked if this "division of labor" could also apply to VAT-resident cDCs. Interestingly, following gene set enrichment analysis (GSEA) pathway analysis we found that the Wnt and PPARγ pathways, which are important for the regulation of adipocyte differentiation and expansion, were selectively upregulated in cDC1 and cDC2, respectively (Figures 1C and 1D) (Christodoulides et al., 2009; Farmer, 2006). The protein expression of active β-catenin was confirmed in Zbtb46-GFP$^{+}$ sorted VAT-cDC1 by western blotting, while PPARγ protein expression was observed in Zbtb46-GFP$^{+}$ VAT-cDC2 (Figure 1E). Interestingly, despite similar gene expression of *Ctnnb1*, *Fzd1*, and *Tcf7/2*, stabilization of β-catenin in splenic cDCs could not be detected (Figure S1D). This suggests that these cDCs upregulate adipocyte-relevant pathways in order to "sense" changes in tissue homeostasis.

Wnt/β-catenin signaling in cDCs has been found to limit inflammatory responses in the intestine, to induce Tregs, and to control Th17 responses (Manicassamy et al., 2010; Suryawanshi et al., 2015). Similarly, PPARγ is known to mediate anti-inflammatory effects of several immune cell types (Ahmadian et al., 2013) and to induce CD4 anergy in bone marrow-derived cDCs (Klotz et al., 2007). To confirm the anti-inflammatory effect of both pathways, we stimulated Zbtb46-GFP$^{+}$ sorted VAT-cDC1 and -cDC2 with a TLR4 agonist (GLA) in the presence or absence of the β-catenin pathway activator SB216763 (SB) or the PPARγ agonist Rosiglitazone (RGZ). In line with previous reports, activation of both pathways suppressed inflammatory responses. However, in concordance with our GSEA results, SB preferentially reduced IL-6 production in cDC1 subset, while RGZ had a significant effect on cDC2 (Figure 2A). A reduction of IL-6 production in cDC2 could be observed after SB treatment that could be a result of residual β-catenin expression in those cells or a non-specific effect of the drug as it did not induce IL-10 production. Interestingly, β-catenin activation significantly enhanced the production of IL-10 from cDC1 from VAT, but not spleen (Figure 2B).

Previous research has established Wnt/β-catenin signaling as an important regulator of adipogenesis. Indeed, local production of the β-catenin agonist WNT10B is known to inhibit pre-adipocyte differentiation (Ross et al., 2000). We confirmed that VAT cells produce WNT10B by ELISA (Figure 2C). Furthermore, recombinant WNT10B was sufficient to induce IL-10 and to suppress GLA-induced IL-6 production by VAT-cDC1 at similar levels to SB (Figures 2D–2F). PPARγ can be activated by dietary lipids, resulting in adipocyte-lipid storage (Wang et al., 2013a). Therefore, both pathways are constitutively activated during regular chow-fed diet in adipocytes/pre-adipocytes but also in tissue-resident cDCs. On this basis, we hypothesized that activation of the Wnt/β-catenin and PPARγ pathway in cDC1 and cDC2, respectively, sustains a tolerogenic phenotype in VAT-DCs and contributes to the maintenance of tissue immune-homeostasis.

### Zbtb46-Cre-Driven Loss of β-Catenin Results in Decreased IL-10 Levels in VAT *In Vivo*

To directly assess the role of these pathways *in vivo*, we crossed floxed β-catenin (*Ctnnb1$^{fl/fl}$*) or PPARγ (*Pparγ$^{fl/fl}$*) mice with transgenic Zbtb46-cre (zDC-cre) mice. This abrogates gene expression specifically in cDCs (Loschko et al., 2016). Unexpectedly, β-catenin deletion was lethal due to Zbtb46$^{+}$ off-target expression in non-hematopoietic cells. To bypass this issue,

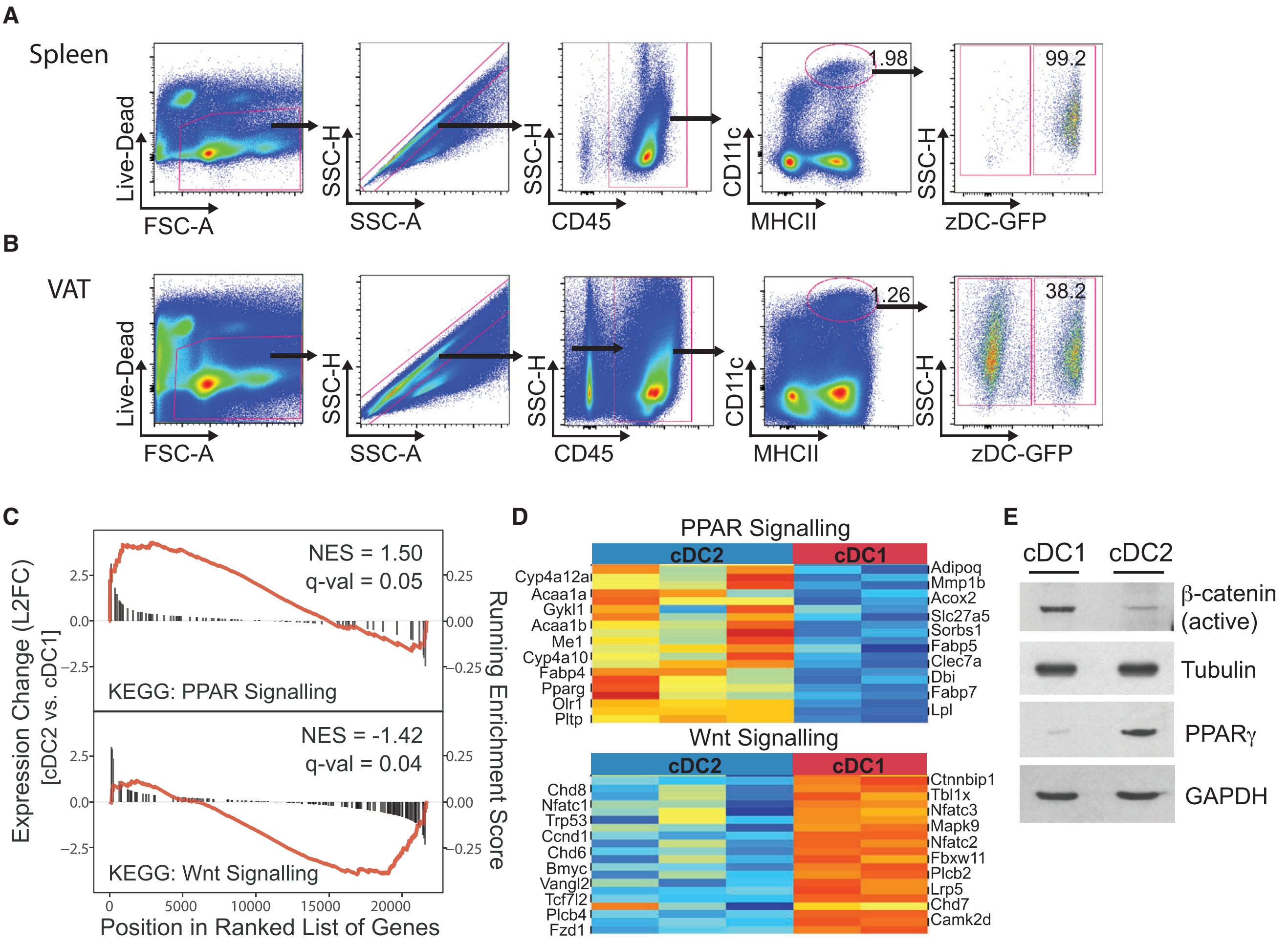


**Figure 1. VAT-cDC Subsets Differentially Upregulate the Wnt/β-Catenin and PPARγ Pathways**

(A and B) Spleen (A) and VAT (B) harvested from lean Zbtb46-GFP mice. cDCs identified as CD11c$^{hi}$ MHCII$^{+}$ GFP$^{+}$ cells by flow cytometry. Dot plots are representative of five independent experiments (n = 15).

(C) GSEA showing significant enrichment of Wnt/β-catenin signature in CD11c$^{hi}$ MHCII$^{+}$ CD11b$^{-}$ cDCs and PPARγ signature in CD11c$^{hi}$ MHCII$^{+}$ CD11b$^{+}$ DCs from VAT (GEO: GSE37448). Graphs represent enrichment plots; x axis is rank order of genes from the most upregulated to the most downregulated between cDC2 and cDC1. Bars highlight the position and log2 fold change (L2FC; y axis, left) of genes in the corresponding gene set; red line indicates running enrichment score (y axis, right). Normalized enrichment score (NES) and p values indicated.

(D) Heatmap of 20 most enriched genes in both pathways.

(E) Expression of active β-catenin and PPARγ detected by western blot representative of three independent experiments.

See also Figure S1.

we generated chimera mice using bone marrows and fetal liver cells from conditional cDC-specific *Pparγ* and *Ctnnb1* knock-down, respectively, to keep experimental settings comparable (chimeric deleted animals are henceforth referred to as *Ctnnb1*$^{-/-}$ or *Pparγ* $^{-/-}$) (Figure S3A). In this model, difference between groups can only be attributed to cDC as zDC-cre is exclusively expressed in cDCs. β-catenin and Pparγ deletion was confirmed by western blotting (Figure S3B). First, we determined if β-catenin and PPARγ signaling pathways are critical for VAT immune homeostasis in steady state. RT-PCR analysis from lean VAT showed significant decrease of IL-10 levels in *Ctnnb1*$^{-/-}$ compared to wild-type (WT) mice, which reached 10-fold decrease in the stromal vascular fraction (SVF) where immune cells are located (Figures 3A and S3C). IL-17 was significant elevated in the SVF (S3C). This was accompanied by a significant decrease of Tregs and a slightly increase, albeit non-significant, of CD4$^{+}$ T cell recruitment to VAT (Figures 3B, S2, and S3D). There were no significant differences in IL-10 levels and T cell recruitment between *Pparγ*$^{-/-}$ and WT mice (Figures 3C, 3D, S3E, and S3F). We hypothesized that this could be the result of two distinct anti-inflammatory mechanisms. While β-catenin prevents the onset of inflammation through IL-10 production, PPARγ exerts its anti-inflammatory effect by interfering with transcriptional regulation of ensuing inflammatory responses, including NF-κβ signaling (Pascual et al., 2005; Ricote et al., 1998). Thus, in the absence of inflammation, the effect of PPARγ is not evident.

While immune cells play a key role in the maintenance of a systemic anti-inflammatory state in lean VAT, it has now become evident that they can also participate in local immune responses.

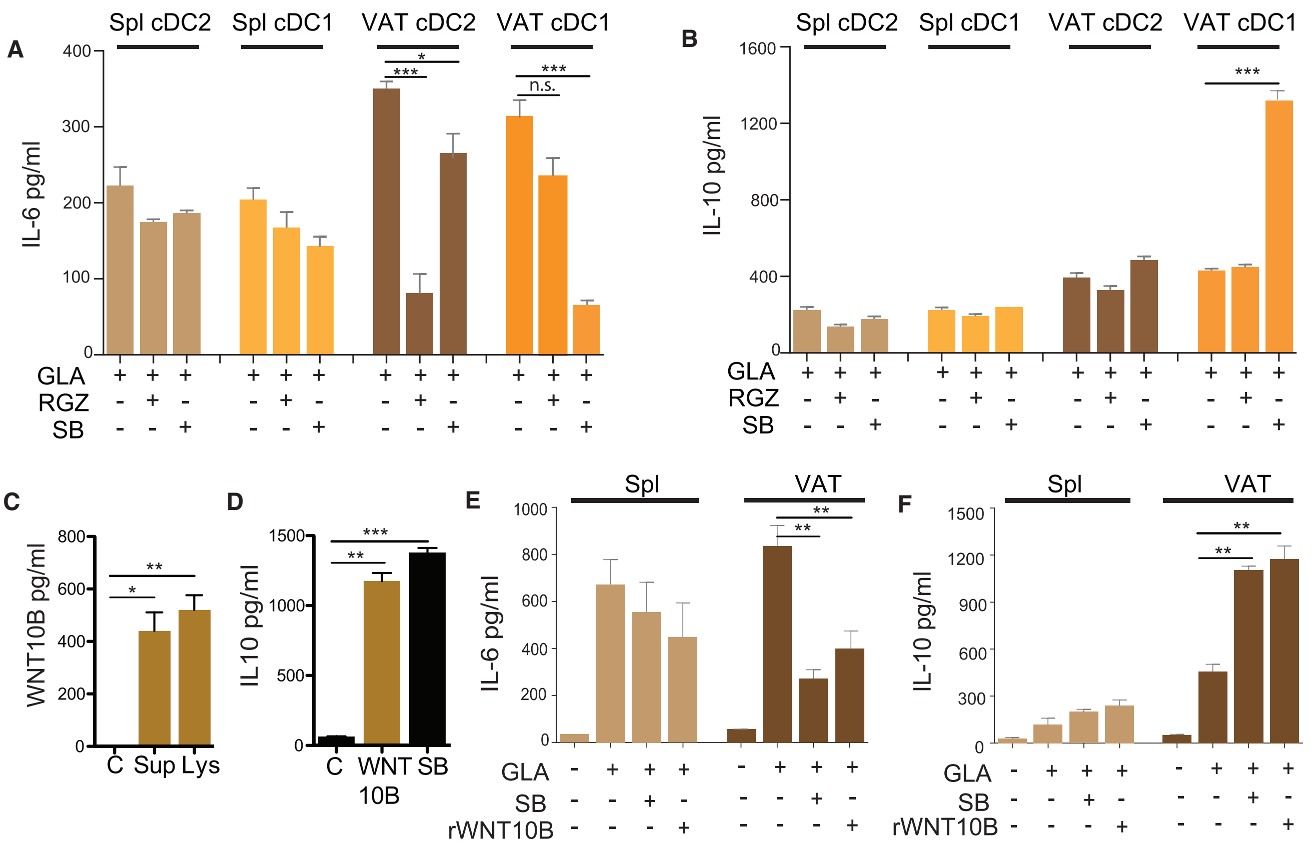


**Figure 2. Activation of the Wnt/β-Catenin and PPARγ Pathway in cDC1 and cDC2 DCs, Respectively, Suppresses TLR4-Induced Inflammation**

cDC1 and cDC2 were purified from spleen and VAT by cell sorting as CD11c$^{hi}$ MHCII$^{+}$ GFP$^{+}$ cells.

(A and B) Cells were incubated overnight with 5 μg GLA in the presence of β-catenin (SB) or PPARγ (RGZ) agonist or DMSO control. IL-6 (A) and IL-10 (B) production in supernatants was measured by ELISA. Bars represent the mean ± SEM and are representative of three independent experiments (n = 9).

(C) A total of 300 μg VAT was isolated from lean mice and cultured overnight (Sup) or immediately lysed (Lys), and Wnt10b production was analyzed by ELISA. Bars represent the mean ± SEM (n = 3).

(D) Total GFP$^{+}$ cDCs were purified and cultured overnight with 100 ng/mL recombinant Wnt10b or SB agonist as positive control; IL-10 was determined in the supernatant by ELISA (n = 9).

(E and F) As described in (D), but in addition, cells were stimulated with GLA in the presence of recombinant Wnt10b; the production of IL-6 (E) and IL-10 (F) was determined in the supernatant by ELISA. Bars represent the mean ± SEM and are representative of three independent experiments (n = 9). Statistical analysis was performed with Student's test, *p < 0.05, **p < 0.01, and ***p < 0.0005.

Indeed, phagocytic cells in VAT or associated immune clusters can collect fluids, pathogens, and cells from the peritoneal cavity and present them to T cells, while cDCs can sample antigens traveling in the collecting lymphatic (Cruz-Migoni and Caamaño, 2016; Kuan et al., 2015). To assess the consequences of β-catenin and PPARγ deletion in VAT-cDC antigen presentation capacity, we immunized mice intraperitoneally with ovalbumin (OVA) after OT-II cell transfer. OVA uptake by phagocytic cells was confirmed by fluorescence microscopy in VAT and flow cytometry in VAT and dLN (Figures 3E, 3F, and S3G). As expected due to their high phagocytic capacity, macrophages have increased OVA uptake (high mean fluorescence). However, as confirmed by many recent studies (Schreiber et al., 2013), macrophages have poor antigen presentation function and failed to induce OT-II proliferation (Figure 3F). OVA uptake could also be confirmed after subcutaneous (s.c.) and intravenous (i.v.) immunization (S3H). Consistent with an anti-inflammatory role of β-catenin signaling in the VAT, only *Ctnnb1*$^{-/-}$ DCs exhibited significantly increased antigen-specific T cell activation, as evidenced by increased OT-II cell division cycles (Figure 3G). This difference was only observed in VAT, but not in LN or spleen (data not shown). Although PPARγ activation in steady-state conditions may not significantly affect VAT immune homeostasis, it may be required to suppress inflammatory responses as observed *in vitro* (Figure 2A). To test this hypothesis, we immunized mice with OVA in the presence of LPS as adjuvant. As expected, the number of dividing OT-II cells and the amount of divisions were increased in *Ctnnb1*$^{-/-}$ mice compared to WT mice, but this time the same effect was observed in *Pparγ*$^{-/-}$ mice in VAT (Figure 3H). Enhanced T cell proliferation was also observed in mesenteric LN (Figure S3I), consistent with a previous report describing increased VAT-DC migration to LN upon inflammation (Kuan et al., 2015). Collectively, these data suggest that, in steady state, β-catenin activation in VAT-cDC1

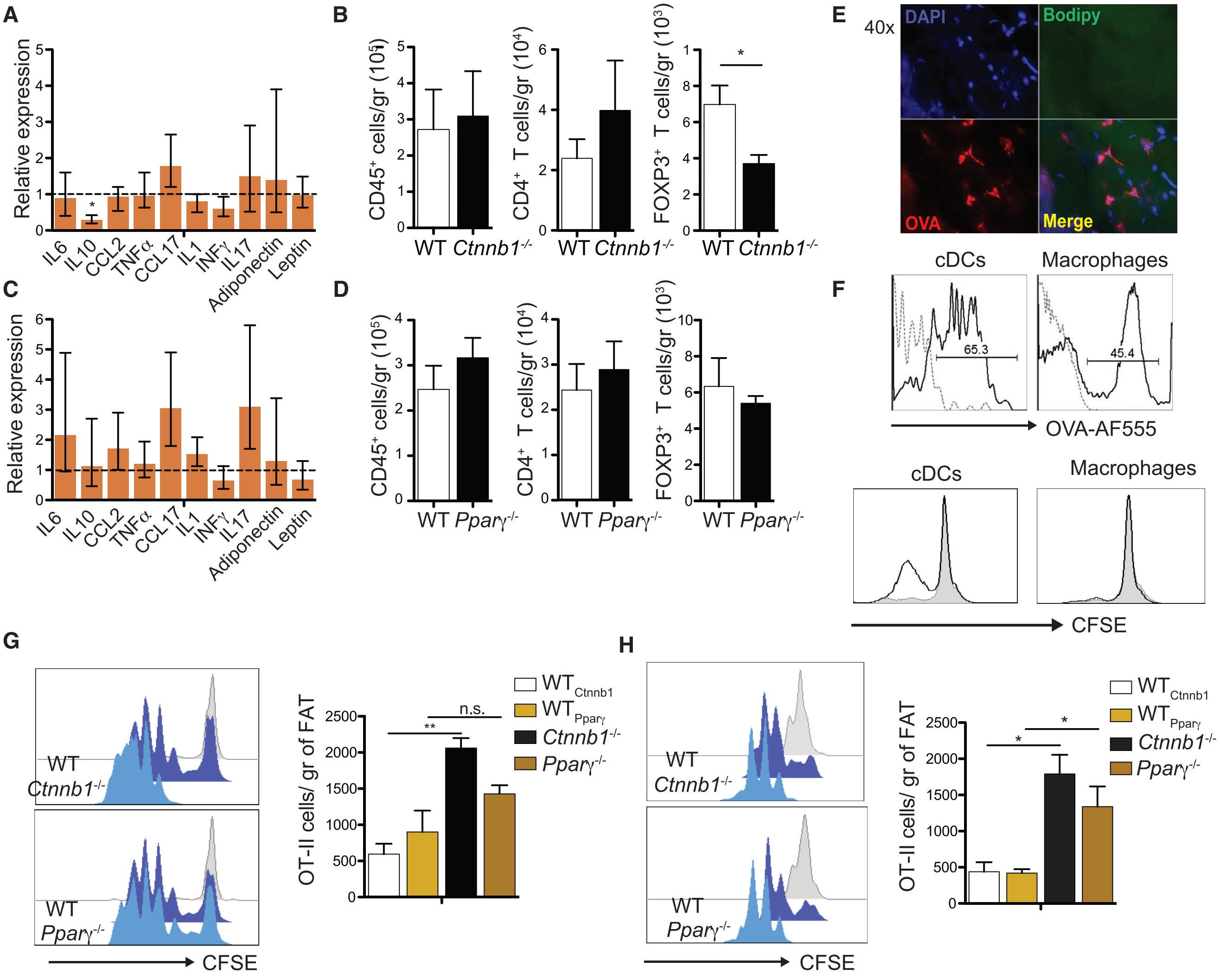


**Figure 3. Deletion of β-Catenin, but Not Pparγ, in cDCs Decreases VAT Anti-inflammatory State in Homeostatic Conditions**

(A) qRT-PCR analysis for gene expression in VAT from lean *Ctnnb1*$^{-/-}$ mice. Expression levels of all genes were normalized against GAPDH RNA. Bars represent mRNA expression on *Ctnnb1*$^{-/-}$ compared to WT mice, which was set as 1 as indicated with dotted line. Error bars indicate the geometric mean of ten biological replicates.

(B) VAT was digested from lean *Ctnnb1*$^{-/-}$ and WT mice. Total numbers of CD45$^{+}$ hematopoietic cells, CD4$^{+}$ T cells, and FoxP3$^{+}$ Tregs were quantified by flow cytometry. Bars represent mean ± SEM (n = 6).

(C) As described in (A) but RT-PCR analysis from lean *Ppar*γ$^{-/-}$ mice.

(D) As described in (B) but graphs represent total cells infiltrated in VAT from lean *Ppar*γ$^{-/-}$ and WT mice. Immune cells were identified as detailed in Figure S2.

(E) Whole-tissue microscopy depicting *in vivo* AF555-labeled ovalbumin (OVA, red) uptake by phagocytic cells in VAT after intraperitoneal (i.p.) immunization. Nuclei labeled by DAPI (blue) and adipocytes by BODIPY (green).

(F) Histograms represent percentage of AF555-OVA uptake of VAT-cDCs (zDC-GFP$^{+}$ CD11c$^{hi}$ MHCII$^{+}$) and VAT-macrophages (MerTK$^{+}$CD64$^{+}$zDC-GFP$^{-}$) after i.p. injection *in vivo*. Mice were injected with OVA. Four hours later, VAT-cDCs and VAT-macrophages were isolated, purified by cell sorting, and cultured with CFSE-labeled OT-II cells *in vitro*. OT-II division was analyzed by flow cytometry. Histograms show CFSE dilution where sample in gray is cells from non-immunized mice.

(G) For *in vivo* antigen presentation, CFSE-labeled OT-II cells were transferred intravenously 1 day before inoculation with 200 μg OVA intraperitoneally. Three days later, cell division (left) and total number (right) of proliferating OT-II T cells were analyzed in VAT from *Ctnnb1*$^{-/-}$, *Ppar*γ$^{-/-}$, and WT mice by flow cytometry. In histogram, samples in gray represent undivided/non-immunized cells. CFSE dilution was analyzed on gated CD45$^{+}$CD3$^{+}$CD4$^{+}$TCRVα2$^{+}$ CFSE$^{+}$ cells.

(H) As described in (G) but mice were immunized with OVA and 1 μg LPS to induce inflammation and OT-II division was analyzed 2 days after immunization. Bars represent mean ± SEM of six mice; dot plots are representative of three independent experiments. Statistical analysis was performed with Student's test, *$p < 0.05$, **$p < 0.01$; n.s., not significant.

See also Figure S3.

is required for IL-10 production, recruitment of Tregs, and the maintenance of an anti-inflammatory state. In addition, IL-10 is known to suppress cDC maturation (Corinti et al., 2001; De Smedt et al., 1997). In contrast, PPARγ stimulation in cDC2 seems to limit VAT-cDC activation under an inflammatory stimulus.

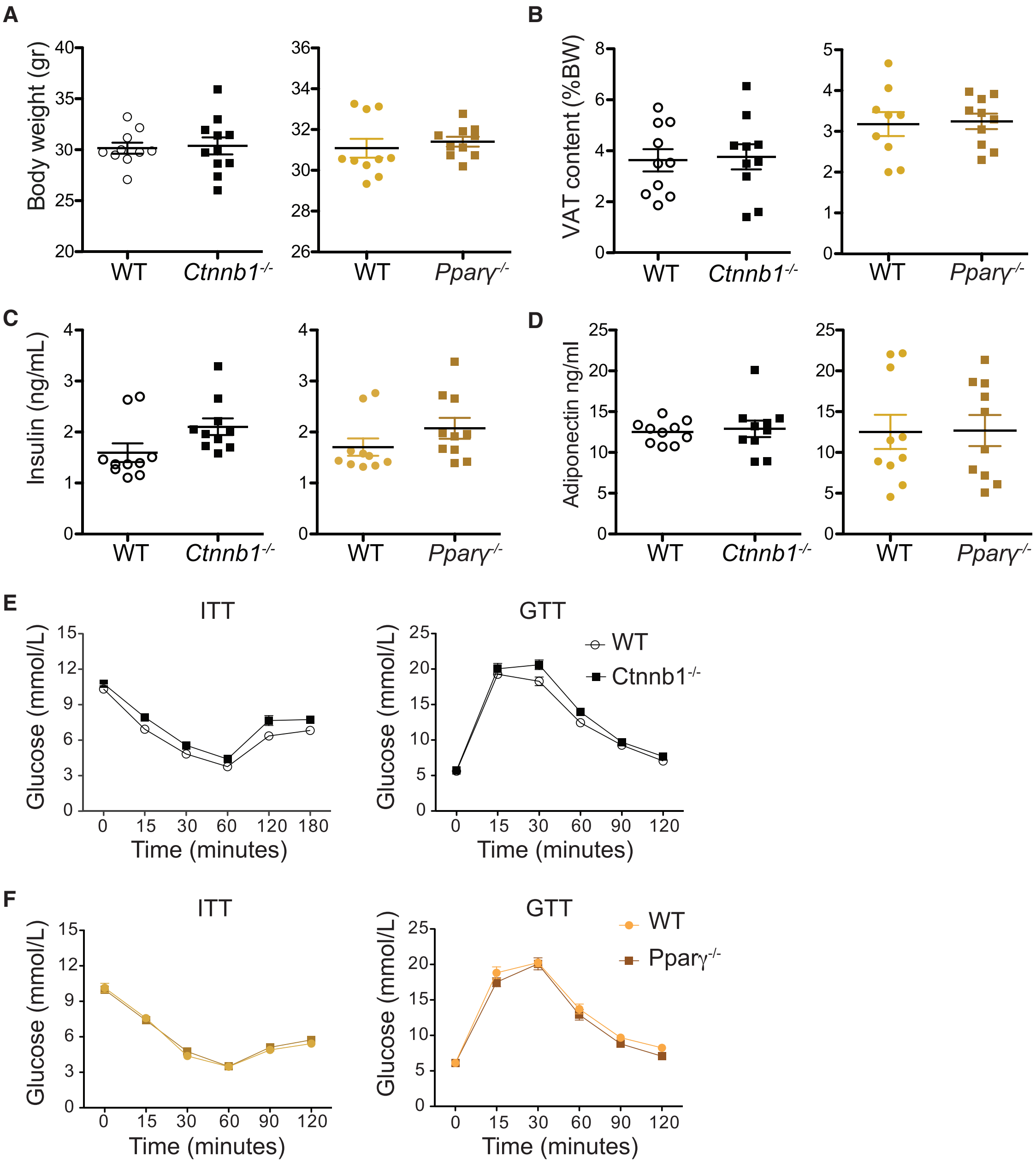


**Figure 4. *Ctnnb1*$^{-/-}$ and *Ppar*$\gamma^{-/-}$ Mice Showed No Statistical Significance in Metabolic Parameters on Chow Diet**

*Ctnnb1*$^{-/-}$ and *Ppar*$\gamma^{-/-}$ mice were fed a chow diet.

(A) Graph shows representative body weights from three independent experiments.

(B) VAT content was calculated as percentage of body weight. Graphs represent the mean ± SEM and are representative of three independent experiments.

(C and D) Fasting insulin (C) and adiponectin (D) were detected by ELISA. Bars represent the mean ± SEM and are representative of three independent experiments. Statistical analysis was performed with Student's test.

(E and F) Whole-body glucose homeostasis measured in (E) *Ctnnb1*$^{-/-}$, (F) *Ppar*$\gamma^{-/-}$, and WT mice on chow diet by i.p. glucose tolerance tests (GTTs; n = 10) and insulin tolerance tests (ITTs; n = 10). Statistical significance at different time points analyzed by two-way ANOVA with Bonferroni's post-test, *$p < 0.05$, **$p < 0.01$, ***$p < 0.001$, and ****$p < 0.0001$.

VAT inflammation has been associated with adipocyte dysfunction, increasing the risk of systemic lipid and glucose metabolic alterations. Despite the signs of reduced VAT anti-inflammatory status in lean conditions, this did not translate into changes in metabolic parameters such as serum insulin and adiponectin levels, weight gain (Figures 4A–4D), or increased insulin resistance as measured by glucose (GTT) and insulin (ITT) tolerance tests (Figures 4E and 4F). This was not surprising since in steady state, VAT inflammation is minimal, local, and easily restrained by the resident anti-inflammatory immune network.

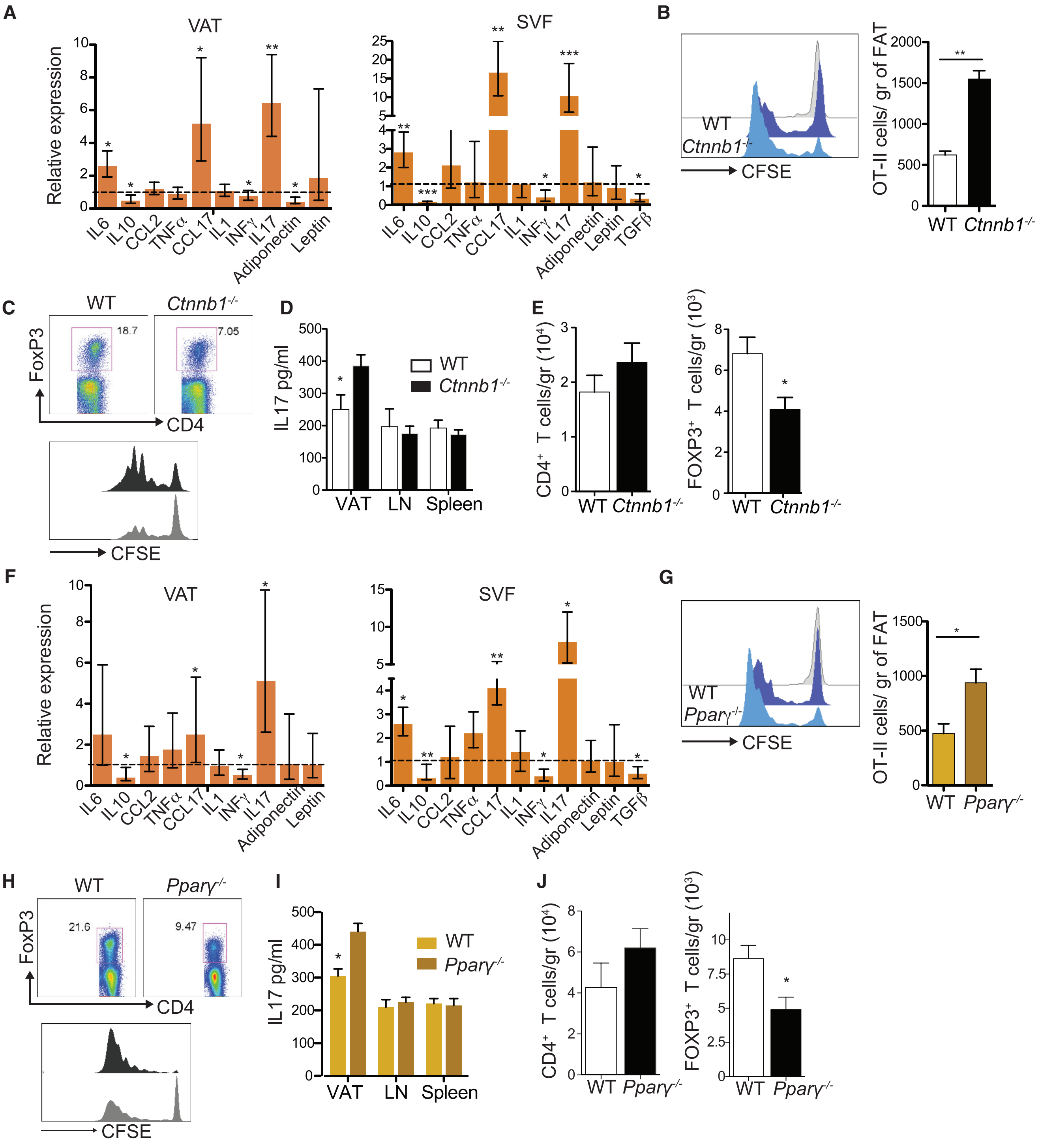


**Figure 5. Deletion of β-Catenin and Pparγ on cDCs Stimulates Pro-inflammatory Responses in VAT upon WD**

Mice were fed a WD for 12 weeks to induce VAT low-grade chronic inflammation.

(A) qRT-PCR analysis for the gene expression in VAT and SVF from *Ctnnb1*$^{-/-}$ and WT control mice on WD (12 weeks). Expression levels of all genes were normalized against GAPDH RNA. Bars represent mRNA expression on *Ctnnb1*$^{-/-}$ compared to WT mice, which was set as 1 as indicated with dotted line. Error bars indicate the geometric mean of ten biological replicates for VAT and five for SVF.

(B) *In vivo* antigen presentation was assessed after CFSE-labeled OT-II transfer and immunization with 200 μg OVA intraperitoneally. Three days later, cell division and total number of proliferating OT-II T cells were analyzed in VAT from *Ctnnb1*$^{-/-}$ and WT mice by flow cytometry. In histogram, samples in gray represent undivided/non-immunized cells. CFSE dilution was analyzed on gated CD45$^{+}$CD3$^{+}$CD4$^{+}$TCRvα2$^{+}$ CFSE$^{+}$ cells.

*(legend continued on next page)*

### cDC-Specific β-Catenin and PPARγ Deletions Exacerbate Obesity-Induced VAT Inflammation and Insulin Resistance

Our data support a complementary role of cDC1 and cDC2 subsets in the regulation of local VAT homeostasis. A great deal of evidence suggests that obesity is associated with low-grade chronic inflammation and that inflammatory responses in VAT play a causal role in obesity-induced insulin resistance in mice and humans (Donath et al., 2013). It is, therefore, tempting to envisage that the anti-inflammatory properties of VAT-DCs may delay the onset of inflammation during chronic over-nutrition. To ascertain the role of β-catenin and PPARγ in cDCs in the control of obesity-induced VAT inflammation, *Ctnnb1*$^{-/-}$ or *Ppar*γ $^{-/-}$ mice were fed a Western diet (WD) for 12 weeks, as well as respective control WT mice. A 10-fold decrease in IL-10 RNA levels in *Ctnnb1*$^{-/-}$ mice was accompanied by significant increase in the T cell chemoattractant CCL17 production and a switch to IL-17 T cell responses in whole VAT as well as the SVF (Figure 5A). In addition, the RNA expression of adiponectin, which is expressed by adipocytes, was reduced, suggesting some degree of adipocyte dysfunction (Figure 5A). Similar to LPS acute inflammation, *in vivo* OT-II proliferation was enhanced in VAT and dLN from *Ctnnb1*$^{-/-}$ compared to WT mice (Figures 5B and S4A). This effect appears to be mediated by a change in cDC activation status since *Ctnnb1*$^{-/-}$ cDCs isolated from the VAT displayed enhanced activation and stimulatory capacity in a mixed-leukocyte reaction (MLR), demonstrating an induction of Th17 T cell responses and reduction of Tregs (Figures 5D and 5E) compared to WT cDCs. Total numbers of VAT-infiltrating CD4$^+$ T cells were slightly increased, albeit not significant, while the numbers of Tregs were significantly reduced (Figure 5E). Accordingly, cDC-derived CCL17 has been shown to promote tissue inflammation by restricting Treg recruitment (Weber et al., 2011). We observed no differences in immune cell composition in dLNs and spleen with the exception of increased numbers of migratory cDCs in dLNs (Figure S4C). Interestingly, *Ppar*γ $^{-/-}$ mice that failed to show differences during steady-state conditions when fed a chow diet displayed a significant increase in T cell-mediated VAT inflammation as evidenced by elevated CCL17 and IL-17 and reduced IL-10 and IFNγ RNA levels compared to WT mice (Figure 5F). Similar to *Ctnnb1*$^{-/-}$, *Ppar*γ $^{-/-}$ cDCs showed *in vivo* increased OVA antigen presentation in VAT and dLN (Figures 5G and S4D) as well as *ex vivo* enhanced stimulatory capacity (MLR) with higher induction of IL-17 CD4$^+$ T cell responses, reduced Treg recruitment (Figures 5H–5J), and increased migration to dLNs (Figure S4D). In this case, we could also detect a significant increase in VAT-infiltrated neutrophils, also evident on chow diet (Figures S4E and S3E), and a decrease in eosinophils, suggesting a more general pro-inflammatory response in the VAT of *Ppar*γ $^{-/-}$ compared to WT mice (Figure S4E).

Based on these observations, we postulated that the increased inflammatory T cell response in VAT of mutant mice could influence whole-body glucose metabolism. β-catenin and PPARγ deletions in cDCs did not affect weight gain, VAT content, or food intake (Figures 6A, S5A, and S5B). No changes were observed in leptin levels, but adiponectin was reduced in *Ctnnb1*$^{-/-}$ compared to WT mice, which confirmed RT-PCR data (Figures 6B and 6C). Liver weight, adipocyte size, and fat deposition in the liver remained unchanged (Figures S5C–S5F). However, cDC-specific *Ctnnb1*$^{-/-}$ and *Ppar*γ $^{-/-}$ mice displayed elevated serum insulin levels compared to WT mice (Figure 6D). In addition, GTTs and ITTs revealed that these mice were significantly less glucose tolerant and more insulin resistant than controls (Figures 6E–6H). In addition, insulin-stimulated AKT phosphorylation was markedly reduced in deficient mice compared to controls in VAT and liver (Figures 6I, 6J, S5G, and S5H). Collectively, these results demonstrate that cDC-specific deletion of β-catenin and PPARγ enhances local inflammatory responses and aggravates obesity-induced insulin resistance.

### VAT Expansion and Inflammation during Long-Term Over-nutrition Curtail β-Catenin and PPARγ Activation in cDC1 and cDC2, Respectively

During obesity, there is evidence to suggest that cDCs adopt an activated phenotype contributing to VAT inflammation. Indeed, chronic over-nutrition promotes cDC recruitment into VAT and increases cDC activation, resulting in enhanced T cell stimulatory capacity and Th17 responses (Figures S6A–S6D). This can be the consequence of (1) overwhelming inflammation that overrides suppressive pathways, (2) inhibition of these pathways in obesity, or (3) both. Current literature strongly supports the last hypothesis. WNT10B levels are suppressed in obesity, allowing adipocyte hyperplasia (Ross et al., 2000). In parallel, upregulation of the protein kinase cyclin-dependent kinase 5 in obesity promotes PPARγ post-transcriptional modifications and inactivation *in vivo* (Choi et al., 2010; Cipolletta et al., 2015). This phenomenon can be replicated with TNFα treatment *in vitro*, providing evidence for an inflammatory origin. Therefore, we asked if these regulatory pathways remain active under chronic over-nutrition. Significant reduction of WNT10B could be confirmed at the transcriptional and protein level (Figures 7A and 7B). WNT10B expression was only marginally reduced in *Ctnnb1*$^{-/-}$ and *Ppar*γ $^{-/-}$ mice, suggesting that it is largely linked to adipocyte differentiation rather than inflammation itself (Figure 7C). As a result, β-catenin activation *in vivo* was reduced (Figure 7D). However, total expression of β-catenin was unaltered (data not shown) and cells were still able to respond to the β-catenin pathway activator SB *ex vivo*, albeit at lower levels, reflecting the activated state of cDCs in VAT (Figures 7E and 7F). Simultaneously, we observed reduced expression of PPARγ on VAT-cDC2 from obese mice (Figure 7G). Accordingly, cDC2 from obese VAT treated with RZG *in vitro* failed to upregulate

(C) *Ex vivo* antigen presentation capacity and activation of VAT-cDCs were evaluated by mixed-leukocyte reactions. T cell proliferation was assessed by CFSE dilution and activation measured by reduced percentages of FoxP3 Treg. Histogram and dot plot are representative of three independent experiments.

(D) IL-17 production by allogeneic T cells was measured in supernatant.

(E) Total numbers of CD4$^+$ T and Treg cells recruited in VAT. Immune cells were identified as detailed in Figure S2.

(F–J) As described in (A)–(G) but VAT inflammation was analyzed in *Ppar*γ$^{-/-}$ mice. Statistical analysis was performed with Student's test, *p < 0.05, **p < 0.01. See also Figure S4.

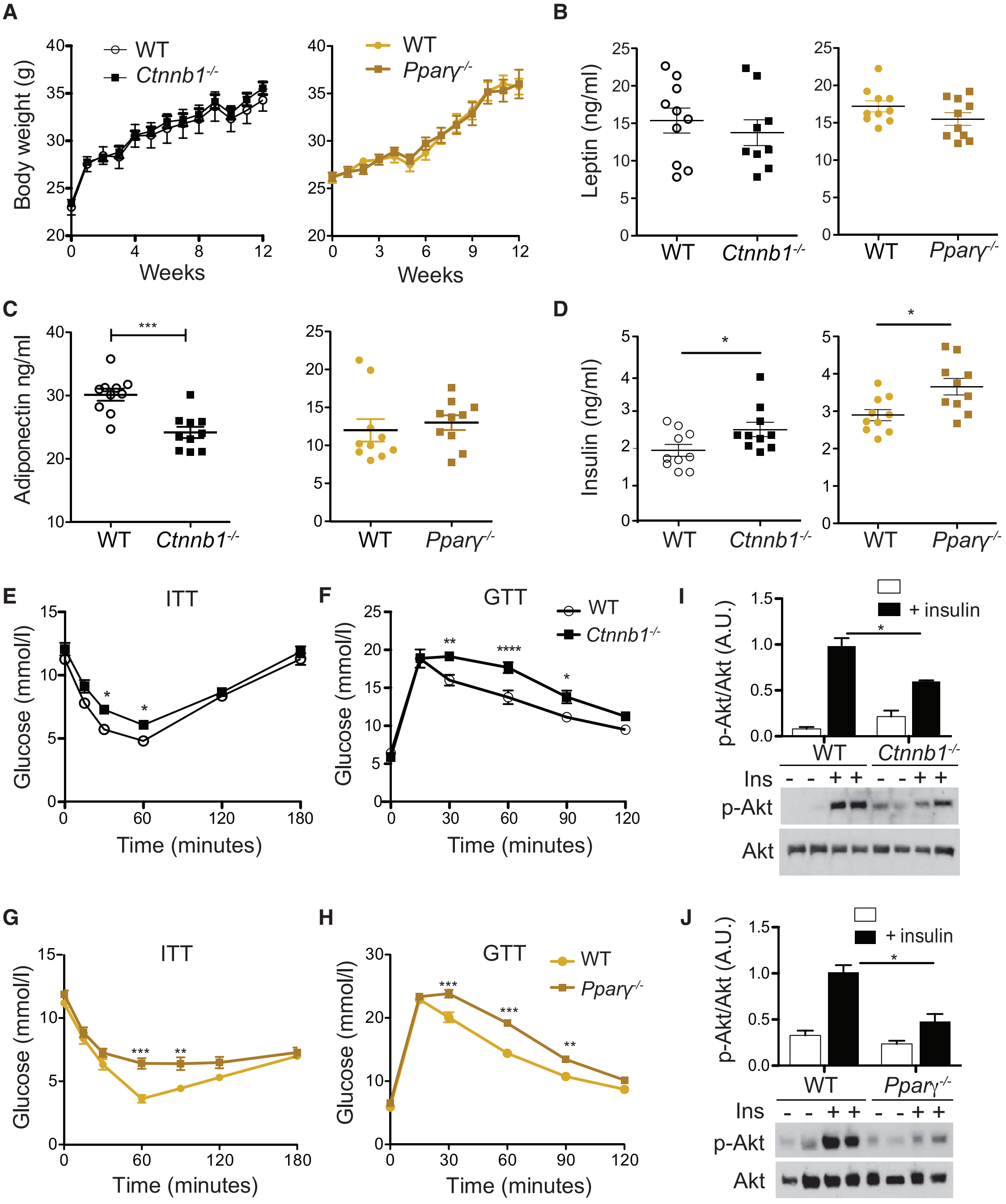


**Figure 6. Deletion of β-Catenin and Pparγ on cDCs Alters Glucose Homeostasis**

(A) Body weight of *Ctnnb1*$^{-/-}$, *Pparγ*$^{-/-}$, and WT mice fed WD for 12 weeks.

(B–D) Serum leptin (B), adiponectin (C), and insulin (D) values from fasted mice representative of two independent experiments (n = 10). Statistical analysis was performed with Student's test.

(E–H) Systemic glucose homeostasis was measured by ITT (E and G) in *Ctnnb1*$^{-/-}$ (E), *Pparγ*$^{-/-}$ (G), and WT control mice fed a WD. Similarly, GTT (F and H) was assessed in *Ctnnb1*$^{-/-}$ (F) and *Pparγ*$^{-/-}$ (H) compared to control mice. Statistical significance at different time points was analyzed by two-way ANOVA with Bonferroni's post-test (n = 10).

(I and J) VAT Akt activation followed acute insulin injection. Western blot analysis of VAT extracts showing phosphor-(Ser473) Akt (p-Akt) levels under control and insulin treatment in *Ctnnb1*$^{-/-}$ (I) and *Pparγ*$^{-/-}$ (J) compared to WT mice. Graphs show densitometry of p-Akt/Akt ratios. *$p < 0.05$, **$p < 0.01$, and ***$p < 0.0005$.

See also Figure S5.

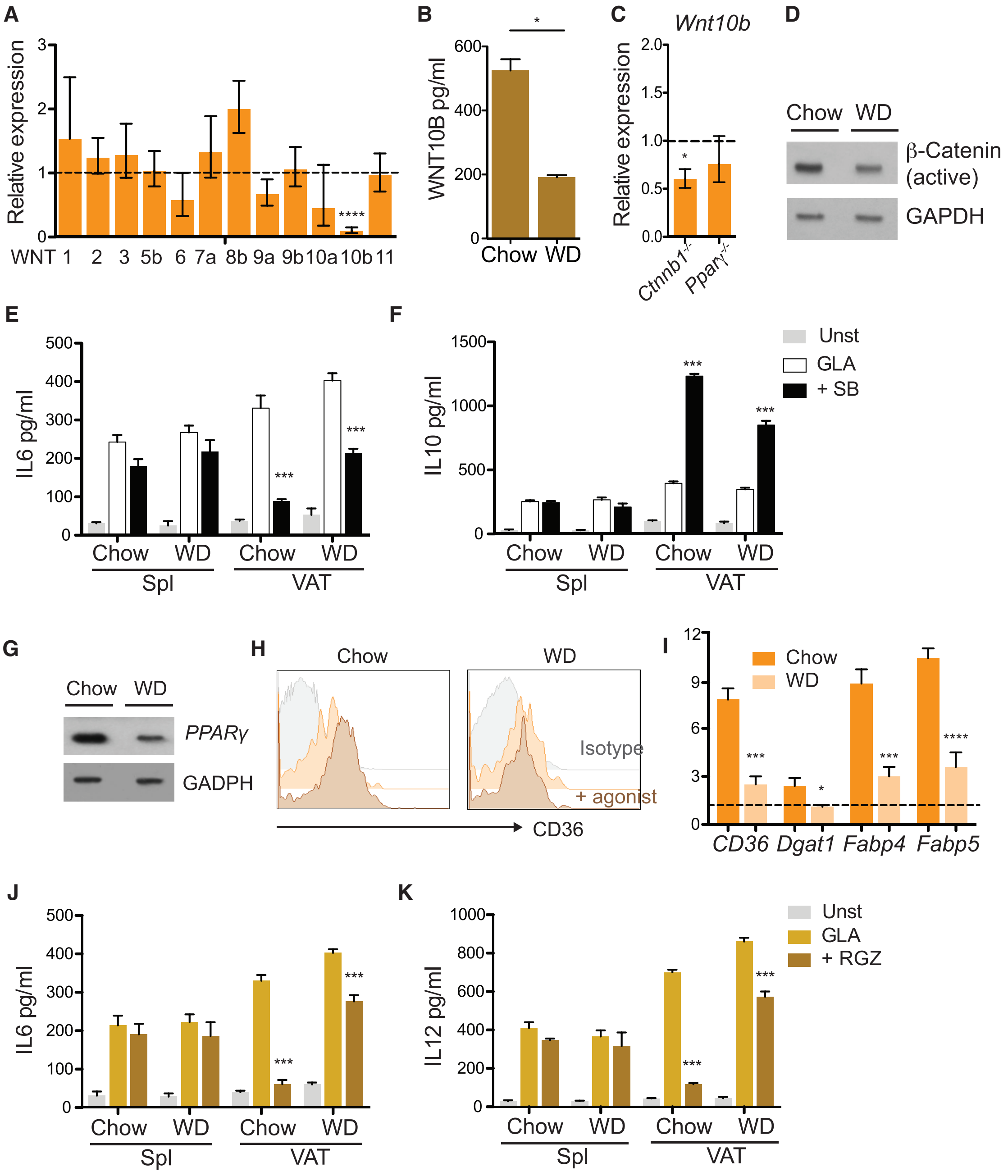


**Figure 7. Chronic Over-nutrition Reduces Wnt/β-Catenin and Pparγ Pathway Activation in VAT-cDCs**

Zbtb46-GFP$^{+}$ mice were fed a chow or WD for 12 weeks.

(A) mRNA expression of Wnt proteins was analyzed by RT-PCR in mice on WD compared to chow diet (12 weeks). Error bars indicate the geometric mean of five biological replicates.

(B) Wnt10b protein levels were detected in VAT homogenates (n = 3).

(C) mRNA expression of Wnt10b in *Ctnnb1*$^{-/-}$ and *Pparγ*$^{-/-}$ mice (n = 10).

(D) Western blot analysis of active β-catenin in purified CD103$^{+}$ Zbtb46-GFP$^{+}$ cDCs from chow- and WD-fed mice.

(E and F) cDC purified from spleen and VAT from Zbtb46-GFP mice were incubated overnight with 5 μg GLA in the presence of β-catenin agonist (SB) or DMSO as control. IL-6 (E) and IL-10 (F) production was measured in supernatants. Bars represent mean ± SD and are representative of two independent experiments (n = 6).

(G) Western blot analysis of PPARγ in purified CD11b$^{+}$ Zbtb46-GFP$^{+}$ cDCs from chow- and WD-fed mice.

(H and I) VAT-cDCs were purified from chow- and WD-fed Zbtb46-GFP$^{+}$ mice. Cells were cultured overnight with PPARγ agonist (RGZ), and CD36 expression was evaluated by flow cytometry (H). Histograms are representative of three independent experiments (H) (n = 3) and mRNA expression of CD36 and other downstream genes (I) (n = 5).

*(legend continued on next page)*

the PPARγ-inducible CD36 and the gene expression of other downstream genes (Figures 7H and 7I), resulting in reduced anti-inflammatory properties as shown by IL-6 and IL-12 production (Figures 7J and 7K). The reduced response to RZG can be solely due to downregulation of PPARγ or to recruitment of newcomer cells. Several reports describe an inhibition of PPARγ signaling *in vivo* (Choi et al., 2010). However, decreased expression of PPARγ was recently observed in adipocytes exposed to free fatty acids (Nguyen et al., 2012). Overall, our findings suggest that chronic over-nutrition partially abrogates the β-catenin and PPARγ anti-inflammatory pathways fueling cDC activation and VAT T cell-mediated inflammation *in vivo*.

## DISCUSSION

cDC function can be dictated by the tissue microenvironment. In this work, we have shown that in order to recognize, integrate, and respond to environmental signals, cDCs must first upregulate pathways relevant for the control of tissue-local homeostasis. In VAT, cDCs are poised to suppress inflammation by activating the β-catenin and PPARγ pathways, which are important regulatory mechanisms for fat expansion. Interestingly, tissue adaptation was cDC subset specific.

The Wnt/β-catenin pathway is evolutionarily conserved and plays a prominent role in cell differentiation, growth, proliferation, survival, and immune cell function (Komiya and Habas, 2008). β-catenin is continuously synthetized but rapidly degraded in the proteasome. The binding of Wnt glycoproteins to Frizzled (Fzd) family receptors in the cell membrane results in the stabilization of β-catenin in the cytoplasm and further translocation into the nucleus, where activation of T cell factor/lymphoid enhancer factor (TCF/LEF) and regulation of gene transcription occur. Several Wnt proteins and Fzd receptors have been described. In VAT, Wnt signaling regulates adipocyte differentiation. Expression of WNT10B is highest in pre-adipocytes and rapidly declines after differentiation. Overexpression of WNT10B stabilizes β-catenin and blocks adipocyte hyperplasia through TCF7/2 activation *in vivo* and *in vitro* (Ross et al., 2000). Similar to cDCs, pre-adipocytes are also located close to adipose vessels, suggesting a close interaction and crosstalk (Tang et al., 2008). Interestingly, VAT-cDC1 showed higher expression of one of the WNT10B receptors, *Fzd1*, and the transcription factor *Tcf7l2*. Activation of β-catenin in cDC1 by WNT10B in the fat induces a tolerogenic phenotype with high production of the anti-inflammatory cytokine IL-10. Conversely, PPARγ is a member of the nuclear hormone receptor superfamily of transcription factors. PPARγ forms a heterodimer with RXR (retinoic X receptor) and subsequently regulates the transcription of cognate target genes involved in lipid and glucose metabolism. PPARγ is highly expressed in VAT, where it is a master regulator of adipogenesis. In mature adipocytes, PPARγ regulates lipid accumulation and hypertrophy (Wang et al., 2013a). We found that PPARγ expression was limited to cDC2 and that its activation repressed inflammatory responses. Several studies have shown an opposite interplay between PPARγ and Wnt/β-catenin signaling within the same cell (Liu et al., 2006). Activation of β-catenin leads to PPARγ repression. Reciprocally, PPARγ inhibits the transcription of β-catenin (Liu et al., 2006). In this context, this differential pathway expression between subsets allows cDCs to simultaneous recognize and respond to both signals in VAT.

The anti-inflammatory role of the Wnt/β-catenin and the PPARγ pathways has been extensively documented *in vitro* and *in vivo*. Indeed, the pathways can protect mice from DSS-induced colitis and obesity-induced insulin resistance, and reduce inflammatory responses during bacterial infections (Gautier et al., 2012; Silva-García et al., 2014). Less is known about their relevance for the control of tissue homeostasis. Here we demonstrate that constitutive activation of β-catenin in VAT-cDC1 promotes an anti-inflammatory milieu under resting conditions. Indeed, the absence of β-catenin in cDCs was sufficient to reduce IL-10 levels and Treg recruitment to VAT rendering the tissue more susceptible to inflammation. By contrast, deletion of PPARγ in Zbtb46-Cre × PPARγ$^{flox/flox}$ mice showed minimal changes in VAT inflammation with some evidence of increased T cell and neutrophil infiltrates, albeit not significant. A possible explanation lies in their different mechanism of action. β-catenin activation triggers PI3K/Akt, which in turns induces IL-10 production (Fu et al., 2015; Manoharan et al., 2014). In contrast, PPARγ can negatively regulate inflammatory gene expression through a mechanism termed transrepression. This pathway operates by preventing the clearance of the repressor complex located on the promoter of inflammatory genes such as NF-kβ and AP-1 (Glass and Ogawa, 2006). Thus, PPARγ acts as a transcriptional repressor of canonical NF-kβ target genes, which are activated upon TLR stimulation, but not in resting conditions (Baratin et al., 2015). Indeed, we observed that IL-6 and T cell stimulatory capacity was elevated in *PPAR*$\gamma^{-/-}$ cDC2 upon TLR4 stimulation.

Chronic VAT inflammation is a common future of obesity and metabolic syndrome. As individual adipocytes enlarge with excess of lipids, they undergo molecular and cellular alterations, resulting in mitochondrial dysfunction, hypoxia, oxidative stress, and eventually cell death. This creates a first local and then systemic sustained inflammatory response altering whole-body glucose homeostasis in mice and men (Donath and Shoelson, 2011; Okin and Medzhitov, 2016). In this context, activation of the Wnt/β-catenin and PPARγ pathways in cDC1 and cDC2, respectively, restrains VAT-T cell recruitment in an attempt to limit the local inflammatory process. Indeed, mice with cDC-specific deletion of β-catenin or PPARγ show increased VAT inflammation associated with altered T cell responses, as evidenced by increased CCL17 and IL-17 and decreased IL-10, IFNγ, and Treg recruitment in VAT from obese mice. Elevated IL-17 production in knockout mice is most likely a consequence of cDC activation as shown by CD11c$^+$ MHCII$^+$ isolated from obese mice. The observed T cell-mediated phenotype, instead of the

(J and K) As described in (E) and (F) but cells were cultured with PPARγ agonist overnight and IL-6 (J) and IL-12 (K) were measured in supernatants. Bars represent mean ± SEM and are representative of two independent experiments. Statistical analysis was performed with Student's test, *$p < 0.05$, **$p < 0.01$, and ***$p < 0.0005$.
See also Figure S6.

innate pro-inflammatory profile in VAT reported upon monocyte/macrophage manipulation, is likely a consequence of the cDC-specific models used in this study, confirming the relevance of cDCs in the control of T cell responses *in vivo* (Odegaard et al., 2007). Increased cumulative inflammation in deficient mice resulted in increased systemic insulin resistance. Changes in adipocyte size and ectopic lipid deposition in liver were minimal; however, *Ctnnb1*$^{-/-}$ mice exhibited reduced VAT and circulating adiponectin, which has anti-inflammatory and cardioprotective functions (den Ruijter et al., 2014). A link between β-catenin signaling and insulin resistance has been previously described. Variants of the *TCF7/2* gene are linked to increased susceptibility for type 2 diabetes (Grant et al., 2006), while a SNP in the human *WNT10b* gene has been associated with early-onset familial obesity (Christodoulides et al., 2006). More compellingly, transgenic mice overexpressing WNT10B in adipocytes resist HFD-induced adipose tissue accumulation (Wright et al., 2007). Paradoxically, despite impaired adipogenesis, WNT10B-overexpressing mice are more glucose tolerant and insulin sensitive, effects attributed partially to reduced VAT inflammation (Wright et al., 2007). Our data suggest an additional mechanism in which activation of β-catenin by WNT10B in cDC1 can contribute to insulin sensitivity in FABP4-WNT10B mice. Similarly, PPARγ agonists, often referred to as an "insulin sensitizer," are potent oral anti-diabetic drugs. The role of PPARγ in VAT immune function is better known. Macrophage expression of PPARγ is required for their polarization toward an anti-inflammatory phenotype, and mice deficient in PPARγ in their macrophage population are more prone to whole-body insulin resistance (Odegaard et al., 2007). In addition, PPARγ activation in Tregs promotes their accumulation in VAT and protection from obesity-induced insulin resistance (Cipolletta et al., 2012). We are now proposing a third immune cell type that responds to PPARγ ligands to reduce VAT inflammation.

Long-term over-nutrition induces profound changes in adipocyte biology that ultimately affect β-catenin and PPARγ signaling. In mice, adipocyte hyperplasia can be observed after 8 weeks of HFD, which translates into reduced WNT10B production by pre-adipocytes. In parallel, PPARγ signaling is impaired due to post-transcriptional modifications (Choi et al., 2010; Cipolletta et al., 2015). Consistent with this observation, we found that activation of β-catenin in cDC1 was partially reduced while PPARγ expression in cDC2 was decreased. The infiltration of PPARγ $^{low}$ cDC2 cannot be ruled out. *In vivo* PPARγ post-transcriptional modification in cDC2 from obese mice requires further investigation. Thus, in the long term, reduction of β-catenin and PPARγ signaling in cDC1 and cDC2, respectively, may explain the inflammatory phenotype observed in VAT-cDCs from obese mice and men (Bertola et al., 2012).

The full implications of our findings are yet to be unraveled. In addition to its metabolic role, VAT is believed to support organ and immune cell functions. Nearly all internal organs and large LN in mammals are embedded in firmly attached adipose tissue. This VAT provides protective "padding" against damage but also serves for the paracrine release of fatty acids and immune mediators for proper tissue function (Iacobellis and Bianco, 2011; Pond and Mattacks, 2003). VAT-cDCs are located in close proximity to lymphatic and blood vessels, where they can sample blood and lymph content. An elegant in-depth study demonstrated that the inherent permeability of collective lymphatic vessels allows fat-resident cDCs to sample soluble antigens trafficking from tissue to LNs. Furthermore, cDCs in the fat were also able to enter the lymphatic vessels and emigrate to adjacent LNs during inflammation (Kuan et al., 2015). Thus, the tolerogenic properties of VAT-cDCs may serve as a checkpoint for the control of tissue inflammation. Under pathological conditions in which lymphatic permeability is compromised, e.g., sustained gut inflammation, chronic lymphedema, and obesity, VAT can be highly infiltrated by antigens leaking out from lymphatic vessels (Fonseca et al., 2015) or can act as a reservoir for viruses, bacteria, and parasites (Damouche et al., 2015; Tanowitz et al., 2017). Therefore, activation of the β-catenin and PPARγ pathways in VAT-cDCs may serve not only to restrain obesity-induced inflammation, but also to promote pathogen persistence and tumor growth. Future studies to investigate these possibilities will be of extreme importance.

## Limitations of Study

During HFD, thorough studies in mice have identified adipocyte hyperplasia in VAT, but not in subcutaneous fat. In addition, the rate of adipocyte hyperplasia is less well understood in human VAT due to technical difficulties. Thus, caution is advised in the extrapolation of these results, in particular the decrease of Wnt10b, to other adipose tissue sites or to human samples.

## STAR★METHODS

Detailed methods are provided in the online version of this paper and include the following:

- KEY RESOURCES TABLE
- CONTACT FOR REAGENT AND RESOURCE SHARING
- EXPERIMENTAL MODEL AND SUBJECT DETAILS
  - Mice
- METHOD DETAILS
  - Real-Time PCR and Expression Profiling
  - *Ex Vivo* Confocal Microscopy
  - Metabolic Assays
  - Flow Cytometry Staining
  - DC Innate Responses
  - DC Antigen Presentation
  - Western Blotting
- QUANTIFICATION AND STATISTICAL ANALYSIS
- DATA AND SOFTWARE AVAILABILITY
  - Data Resources

## SUPPLEMENTAL INFORMATION

Supplemental Information includes six figures and one table and can be found with this article online at https://doi.org/10.1016/j.cmet.2018.02.007.

## ACKNOWLEDGMENTS

This work is dedicated to the memory of Ralph Steinman and Cheolho Cheong. We are indebted to M. Nussenzweig (The Rockefeller University) for providing the Zbtb46-Cre mice. We thank Joanne Smith for technical support and members of the Marelli-Berg lab at QMUL for scientific discussions. This work was supported by the British Heart Foundation FS/13/49/30421 (M.P.L.), PG/16/79/32419 (M.P.L. and E.G.W.), and FS/14/66/31293 (C.E.M.), and Marie Curie/Cascade CF-2013-11-003-longhi (M.P.L.). N.F. and M.E.R. are funded

by Action Against Cancer and the Hilary Craft Foundation (PS8508) and M.C. by the MRC (MR/L002345/1) and the Medical College of Saint Bartholomew's Hospital Trust. F.C.C. is funded by a studentship from Bart's and the London School of Medicine.

## AUTHOR CONTRIBUTIONS

M.P.L. conceived the study; C.E.M., E.G.W., M.C., C.G.-M., and M.P.L. designed experiments; C.E.M., E.G.W., M.-B.V., and M.P.L. performed and analyzed experiments; J.L. generated the Zbtb46-Cre strain; V.S. and F.C.C. provided technical support; M.E.R., L.C., and N.F. performed informatics analysis; F.M.-B. contributed intellectually; C.E.M. and E.G.W. helped with manuscript preparation; and M.P.L. wrote the manuscript.

## DECLARATION OF INTERESTS

The authors declare no competing interests.

Received: September 8, 2017
Revised: December 20, 2017
Accepted: February 2, 2018
Published: March 6, 2018

## REFERENCES

Ahmadian, M., Suh, J.M., Hah, N., Liddle, C., Atkins, A.R., Downes, M., and Evans, R.M. (2013). PPARγ signaling and metabolism: the good, the bad and the future. Nat. Med. *19*, 557–566.

Arokiasamy, S., Zakian, C., Dilliway, J., Wang, W., Nourshargh, S., and Voisin, M.B. (2017). Endogenous TNFα orchestrates the trafficking of neutrophils into and within lymphatic vessels during acute inflammation. Sci. Rep. *7*, 44189.

Baratin, M., Foray, C., Demaria, O., Habbeddine, M., Pollet, E., Maurizio, J., Verthuy, C., Davanture, S., Azukizawa, H., Flores-Langarica, A., et al. (2015). Homeostatic NF-κB signaling in steady-state migratory dendritic cells regulates immune homeostasis and tolerance. Immunity *42*, 627–639.

Bertola, A., Ciucci, T., Rousseau, D., Bourlier, V., Duffaut, C., Bonnafous, S., Blin-Wakkach, C., Anty, R., Iannelli, A., Gugenheim, J., et al. (2012). Identification of adipose tissue dendritic cells correlated with obesity-associated insulin-resistance and inducing Th17 responses in mice and patients. Diabetes *61*, 2238–2247.

Chawla, A., Nguyen, K.D., and Goh, Y.P. (2011). Macrophage-mediated inflammation in metabolic disease. Nat. Rev. Immunol. *11*, 738–749.

Choi, J.H., Banks, A.S., Estall, J.L., Kajimura, S., Boström, P., Laznik, D., Ruas, J.L., Chalmers, M.J., Kamenecka, T.M., Blüher, M., et al. (2010). Anti-diabetic drugs inhibit obesity-linked phosphorylation of PPARgamma by Cdk5. Nature *466*, 451–456.

Christodoulides, C., Scarda, A., Granzotto, M., Milan, G., Dalla Nora, E., Keogh, J., De Pergola, G., Stirling, H., Pannacciulli, N., Sethi, J.K., et al. (2006). WNT10B mutations in human obesity. Diabetologia *49*, 678–684.

Christodoulides, C., Lagathu, C., Sethi, J.K., and Vidal-Puig, A. (2009). Adipogenesis and WNT signalling. Trends Endocrinol. Metab. *20*, 16–24.

Cipolletta, D., Feuerer, M., Li, A., Kamei, N., Lee, J., Shoelson, S.E., Benoist, C., and Mathis, D. (2012). PPAR-γ is a major driver of the accumulation and phenotype of adipose tissue Treg cells. Nature *486*, 549–553.

Cipolletta, D., Cohen, P., Spiegelman, B.M., Benoist, C., and Mathis, D. (2015). Appearance and disappearance of the mRNA signature characteristic of Treg cells in visceral adipose tissue: age, diet, and PPARγ effects. Proc. Natl. Acad. Sci. USA *112*, 482–487.

Corinti, S., Albanesi, C., la Sala, A., Pastore, S., and Girolomoni, G. (2001). Regulatory activity of autocrine IL-10 on dendritic cell functions. J. Immunol. *166*, 4312–4318.

Cruz-Migoni, S., and Caamaño, J. (2016). Fat-associated lymphoid clusters in inflammation and immunity. Front. Immunol. *7*, 612.

Damouche, A., Lazure, T., Avettand-Fènoël, V., Huot, N., Dejucq-Rainsford, N., Satie, A.P., Mélard, A., David, L., Gommet, C., Ghosn, J., et al. (2015). Adipose tissue is a neglected viral reservoir and an inflammatory site during chronic HIV and SIV infection. PLoS Pathog. *11*, e1005153.

De Smedt, T., Van Mechelen, M., De Becker, G., Urbain, J., Leo, O., and Moser, M. (1997). Effect of interleukin-10 on dendritic cell maturation and function. Eur. J. Immunol. *27*, 1229–1235.

den Ruijter, H.M., Pasterkamp, G., and de Jager, S.C. (2014). Adiponectin regulation in cardiovascular disease: is diseased fat showing its true color? Arterioscler. Thromb. Vasc. Biol. *34*, 2180–2181.

Donath, M.Y., and Shoelson, S.E. (2011). Type 2 diabetes as an inflammatory disease. Nat. Rev. Immunol. *11*, 98–107.

Donath, M.Y., Dalmas, É., Sauter, N.S., and Böni-Schnetzler, M. (2013). Inflammation in obesity and diabetes: islet dysfunction and therapeutic opportunity. Cell Metab. *17*, 860–872.

Farmer, S.R. (2006). Transcriptional control of adipocyte formation. Cell Metab. *4*, 263–273.

Fonseca, D.M., Hand, T.W., Han, S.J., Gerner, M.Y., Glatman Zaretsky, A., Byrd, A.L., Harrison, O.J., Ortiz, A.M., Quinones, M., Trinchieri, G., et al. (2015). Microbiota-dependent sequelae of acute infection compromise tissue-specific immunity. Cell *163*, 354–366.

Fu, C., Liang, X., Cui, W., Ober-Blöbaum, J.L., Vazzana, J., Shrikant, P.A., Lee, K.P., Clausen, B.E., Mellman, I., and Jiang, A. (2015). β-catenin in dendritic cells exerts opposite functions in cross-priming and maintenance of CD8+ T cells through regulation of IL-10. Proc. Natl. Acad. Sci. USA *112*, 2823–2828.

Galarraga, M., Campión, J., Muñoz-Barrutia, A., Boqué, N., Moreno, H., Martínez, J.A., Milagro, F., and Ortiz-de-Solórzano, C. (2012). Adiposoft: automated software for the analysis of white adipose tissue cellularity in histological sections. J. Lipid Res. *53*, 2791–2796.

Gaskill, B.N., Gordon, C.J., Pajor, E.A., Lucas, J.R., Davis, J.K., and Garner, J.P. (2012). Heat or insulation: behavioral titration of mouse preference for warmth or access to a nest. PLoS One *7*, e32799.

Gautier, E.L., Chow, A., Spanbroek, R., Marcelin, G., Greter, M., Jakubzick, C., Bogunovic, M., Leboeuf, M., van Rooijen, N., Habenicht, A.J., et al. (2012). Systemic analysis of PPARγ in mouse macrophage populations reveals marked diversity in expression with critical roles in resolution of inflammation and airway immunity. J. Immunol. *189*, 2614–2624.

Glass, C.K., and Ogawa, S. (2006). Combinatorial roles of nuclear receptors in inflammation and immunity. Nat. Rev. Immunol. *6*, 44–55.

Grant, S.F., Thorleifsson, G., Reynisdottir, I., Benediktsson, R., Manolescu, A., Sainz, J., Helgason, A., Stefansson, H., Emilsson, V., Helgadottir, A., et al. (2006). Variant of transcription factor 7-like 2 (TCF7L2) gene confers risk of type 2 diabetes. Nat. Genet. *38*, 320–323.

Iacobellis, G., and Bianco, A.C. (2011). Epicardial adipose tissue: emerging physiological, pathophysiological and clinical features. Trends Endocrinol. Metab. *22*, 450–457.

Klotz, L., Dani, I., Edenhofer, F., Nolden, L., Evert, B., Paul, B., Kolanus, W., Klockgether, T., Knolle, P., and Diehl, L. (2007). Peroxisome proliferator-activated receptor gamma control of dendritic cell function contributes to development of CD4+ T cell anergy. J. Immunol. *178*, 2122–2131.

Komiya, Y., and Habas, R. (2008). Wnt signal transduction pathways. Organogenesis *4*, 68–75.

Kuan, E.L., Ivanov, S., Bridenbaugh, E.A., Victora, G., Wang, W., Childs, E.W., Platt, A.M., Jakubzick, C.V., Mason, R.J., Gashev, A.A., et al. (2015). Collecting lymphatic vessel permeability facilitates adipose tissue inflammation and distribution of antigen to lymph node-homing adipose tissue dendritic cells. J. Immunol. *194*, 5200–5210.

Liu, J., Wang, H., Zuo, Y., and Farmer, S.R. (2006). Functional interaction between peroxisome proliferator-activated receptor gamma and beta-catenin. Mol. Cell. Biol. *26*, 5827–5837.

Loschko, J., Schreiber, H.A., Rieke, G.J., Esterházy, D., Meredith, M.M., Pedicord, V.A., Yao, K.H., Caballero, S., Pamer, E.G., Mucida, D., and Nussenzweig, M.C. (2016). Absence of MHC class II on cDCs results in microbial-dependent intestinal inflammation. J. Exp. Med. *213*, 517–534.

Manicassamy, S., Reizis, B., Ravindran, R., Nakaya, H., Salazar-Gonzalez, R.M., Wang, Y.C., and Pulendran, B. (2010). Activation of beta-catenin in

dendritic cells regulates immunity versus tolerance in the intestine. Science *329*, 849–853.

Manoharan, I., Hong, Y., Suryawanshi, A., Angus-Hill, M.L., Sun, Z., Mellor, A.L., Munn, D.H., and Manicassamy, S. (2014). TLR2-dependent activation of β-catenin pathway in dendritic cells induces regulatory responses and attenuates autoimmune inflammation. J. Immunol. *193*, 4203–4213.

Mattacks, C.A., Sadler, D., and Pond, C.M. (2004). The effects of dietary lipids on dendritic cells in perinodal adipose tissue during chronic mild inflammation. Br. J. Nutr. *91*, 883–892.

Menezes, S., Melandri, D., Anselmi, G., Perchet, T., Loschko, J., Dubrot, J., Patel, R., Gautier, E.L., Hugues, S., Longhi, M.P., et al. (2016). The heterogeneity of Ly6Chi monocytes controls their differentiation into iNOS+ macrophages or monocyte-derived dendritic cells. Immunity *45*, 1205–1218.

Merad, M., Sathe, P., Helft, J., Miller, J., and Mortha, A. (2013). The dendritic cell lineage: ontogeny and function of dendritic cells and their subsets in the steady state and the inflamed setting. Annu. Rev. Immunol. *31*, 563–604.

Nguyen, M.T., Chen, A., Lu, W.J., Fan, W., Li, P.P., Oh, D.Y., and Patsouris, D. (2012). Regulation of chemokine and chemokine receptor expression by PPARγ in adipocytes and macrophages. PLoS One *7*, e34976.

Odegaard, J.I., Ricardo-Gonzalez, R.R., Goforth, M.H., Morel, C.R., Subramanian, V., Mukundan, L., Red Eagle, A., Vats, D., Brombacher, F., Ferrante, A.W., and Chawla, A. (2007). Macrophage-specific PPARgamma controls alternative activation and improves insulin resistance. Nature *447*, 1116–1120.

Okin, D., and Medzhitov, R. (2016). The effect of sustained inflammation on hepatic mevalonate pathway results in hyperglycemia. Cell *165*, 343–356.

Pascual, G., Fong, A.L., Ogawa, S., Gamliel, A., Li, A.C., Perissi, V., Rose, D.W., Willson, T.M., Rosenfeld, M.G., and Glass, C.K. (2005). A SUMOylation-dependent pathway mediates transrepression of inflammatory response genes by PPAR-gamma. Nature *437*, 759–763.

Patsouris, D., Li, P.P., Thapar, D., Chapman, J., Olefsky, J.M., and Neels, J.G. (2008). Ablation of CD11c-positive cells normalizes insulin sensitivity in obese insulin resistant animals. Cell Metab. *8*, 301–309.

Pond, C.M., and Mattacks, C.A. (2003). The source of fatty acids incorporated into proliferating lymphoid cells in immune-stimulated lymph nodes. Br. J. Nutr. *89*, 375–383.

R Core Team. (2016). R: a language and environment for statistical computing (Vienna, Austria).

Ricote, M., Li, A.C., Willson, T.M., Kelly, C.J., and Glass, C.K. (1998). The peroxisome proliferator-activated receptor-gamma is a negative regulator of macrophage activation. Nature *391*, 79–82.

Ritchie, M.E., Phipson, B., Wu, D., Hu, Y., Law, C.W., Shi, W., and Smyth, G.K. (2015). limma powers differential expression analyses for RNA-sequencing and microarray studies. Nucleic Acids Res. *43*, e47.

Ross, S.E., Hemati, N., Longo, K.A., Bennett, C.N., Lucas, P.C., Erickson, R.L., and MacDougald, O.A. (2000). Inhibition of adipogenesis by Wnt signaling. Science *289*, 950–953.

Schipper, H.S., Prakken, B., Kalkhoven, E., and Boes, M. (2012). Adipose tissue-resident immune cells: key players in immunometabolism. Trends Endocrinol. Metab. *23*, 407–415.

Schreiber, H.A., Loschko, J., Karssemeijer, R.A., Escolano, A., Meredith, M.M., Mucida, D., Guermonprez, P., and Nussenzweig, M.C. (2013). Intestinal monocytes and macrophages are required for T cell polarization in response to Citrobacter rodentium. J. Exp. Med. *210*, 2025–2039.

Silva-García, O., Valdez-Alarcón, J.J., and Baizabal-Aguirre, V.M. (2014). The Wnt/β-catenin signaling pathway controls the inflammatory response in infections caused by pathogenic bacteria. Mediators Inflamm. *2014*, 310183.

Stefanovic-Racic, M., Yang, X., Turner, M.S., Mantell, B.S., Stolz, D.B., Sumpter, T.L., Sipula, I.J., Dedousis, N., Scott, D.K., Morel, P.A., et al. (2012). Dendritic cells promote macrophage infiltration and comprise a substantial proportion of obesity-associated increases in CD11c+ cells in adipose tissue and liver. Diabetes *61*, 2330–2339.

Suryawanshi, A., Manoharan, I., Hong, Y., Swafford, D., Majumdar, T., Taketo, M.M., Manicassamy, B., Koni, P.A., Thangaraju, M., Sun, Z., et al. (2015). Canonical wnt signaling in dendritic cells regulates Th1/Th17 responses and suppresses autoimmune neuroinflammation. J. Immunol. *194*, 3295–3304.

Tang, W., Zeve, D., Suh, J.M., Bosnakovski, D., Kyba, M., Hammer, R.E., Tallquist, M.D., and Graff, J.M. (2008). White fat progenitor cells reside in the adipose vasculature. Science *322*, 583–586.

Tanowitz, H.B., Scherer, P.E., Mota, M.M., and Figueiredo, L.M. (2017). Adipose tissue: a safe haven for parasites? Trends Parasitol. *33*, 276–284.

Wang, F., Mullican, S.E., DiSpirito, J.R., Peed, L.C., and Lazar, M.A. (2013a). Lipoatrophy and severe metabolic disturbance in mice with fat-specific deletion of PPARγ. Proc. Natl. Acad. Sci. USA *110*, 18656–18661.

Wang, Q.A., Tao, C., Gupta, R.K., and Scherer, P.E. (2013b). Tracking adipogenesis during white adipose tissue development, expansion and regeneration. Nat. Med. *19*, 1338–1344.

Weber, C., Meiler, S., Döring, Y., Koch, M., Drechsler, M., Megens, R.T., Rowinska, Z., Bidzhekov, K., Fecher, C., Ribechini, E., et al. (2011). CCL17-expressing dendritic cells drive atherosclerosis by restraining regulatory T cell homeostasis in mice. J. Clin. Invest. *121*, 2898–2910.

Wright, W.S., Longo, K.A., Dolinsky, V.W., Gerin, I., Kang, S., Bennett, C.N., Chiang, S.H., Prestwich, T.C., Gress, C., Burant, C.F., et al. (2007). Wnt10b inhibits obesity in ob/ob and agouti mice. Diabetes *56*, 295–303.

# STAR★METHODS

## KEY RESOURCES TABLE

| REAGENT or RESOURCE | SOURCE | IDENTIFIER |
|---|---|---|
| Antibodies | | |
| Anti-Mouse CD16/32-FITC (clone 93) | BioLegend | RRID: AB_312805 |
| Anti-Mouse F4/80-PerCP (clone BM8) | BioLegend | RRID: AB_893484 |
| Anti-Mouse CD206-BV421 (clone C068C2) | BioLegend | RRID: AB_2562232 |
| Anti-Mouse CD11c-BV605 (clone HL3) | BD Biosciences | Cat#563057 |
| Anti-Mouse CD11c-BV605 (clone N418) | BioLegend | RRID: AB_2562415 |
| Anti-Mouse CD103-APC (clone2E7) | eBioscience | RRID: AB_1106992 |
| Anti-Mouse MHCII-AF700 (clone M5/114.15.2) | BioLegend | RRID: AB_493727 |
| Anti-Mouse CD11b-AF780 (clone M1/70) | eBioscience | RRID: AB_1603193 |
| Anti-Mouse MertK-PE | R&D Systems | Cat#FAB5912P |
| Anti-Mouse CD45-PE-CF594 (clone 30-F11) | BioLegend | RRID: AB_2564003 |
| Anti-Mouse CD45-PE-CF594 (clone 30-F11) | BD Biosciences | RRID: AB_11154401 |
| Anti-Mouse CD64-PE-Cy7 (clone X54-5/7.1) | BioLegend | RRID: AB_2563904 |
| Anti-Mouse CD8-FITC (clone 53-6.7) | eBioscience | RRID: AB_464916 |
| Anti-Mouse Ly6G-BV421 (clone IA8) | BioLegend | RRID: AB_2562567 |
| Anti-Mouse NK1.1-BV605 (clone PK136) | BioLegend | RRID: AB_2562274 |
| Anti-Mouse CD3-AF700 (clone 17A2) | eBioscience | RRID: AB_529508 |
| Anti-Mouse SiglecF-PE-CF594 (clone E50-2440) | BD Biosciences | RRID: AB_2687994 |
| Anti-Mouse CD4-PE-Cy7 (clone RM4-5) | BioLegend | RRID: AB_312729 |
| Anti-Mouse B220-PerCP (clone RA3-6B2) | BioLegend | RRID: AB_893354 |
| Anti-Mouse FOXP3-PE (clone 150D/E4) | eBioscience | RRID: AB_10670338 |
| Anti-Mouse CD36-APC (clone HM36) | BioLegend | RRID: AB_2072639 |
| Anti-Mouse CD86-BV605 (clone GL-1) | BioLegend | RRID: AB_11204429 |
| Anti-Mouse CD80-BV (clone 3H5) | BD Biosciences | RRID: AB_395646 |
| Anti-Mouse TCRVα2-PE (clone B20.1) | BioLegend | RRID: AB_1134183 |
| Non-phospho (Active) β-Catenin Rabbit mAb (clone D13A1) | Cell Signaling Technologies | RRID: AB_11127203 |
| PPARγ Rabbit mAb (clone C26H12) | Cell Signaling Technologies | RRID: AB_2166051 |
| Mouse Tubulin mAb (clone GT114) | GeneTex | RRID: AB_2716636 |
| Mouse GAPDH mAb (clone GT239) | GeneTex | RRID: AB_11174761 |
| Phospho-Akt Rabbit mAb (Ser473) (clone 193H12) | Cell Signaling Technologies | RRID: AB_331168 |
| Akt Rabbit mAb | Cell Signaling Technologies | RRID: AB_329827 |
| Anti-mouse IgG, HRP linked (from sheep) | GE Healthcare Life Sciences | Cat#NA931V |
| Anti-rabbit IgG, HRP linked (from donkey) | GE Healthcare Life Sciences | Cat#NA934V |
| Anti-Mouse LYVE-1 (clone ALY7) | eBioscience | RRID: AB_1633414 |
| Anti-Mouse PECAM-1 (clone 390) | eBioscience | RRID: AB_467201 |
| Chemicals, Peptides, and Recombinant Proteins | | |
| TRIzol Reagent | Invitrogen | Cat#15596026 |
| Collagenase D | Roche | Cat# 11088882001 |
| Collagenase Type II | Sigma | Cat#C6885 |
| DNaseI | Sigma | Cat#D4527 |
| GLA | IDRI | Cat#EM082 |
| Rosiglitazone | Sigma | Cat#R2408 |
| SB216763 | Abcam Biochemicals | Cat# ab120202 |
| Recombinant Wnt10b | R&D Systems | Cat#2110-WN-010 |
| CFSE | Sigma | Cat#21888 |

(*Continued on next page*)

| Continued | | |
|---|---|---|
| REAGENT or RESOURCE | SOURCE | IDENTIFIER |
| Ovalbumin | Sigma | Cat#S7951 |
| LPS | Sigma | Cat#L6143 |
| Ovalbumin- Alexa Fluor 555 | Invitrogen | Cat#034782 |
| BODIPY FL C16 | Invitrogen | Cat#D3821 |
| Critical Commercial Assays | | |
| RNeasy MiniElute cleanup kit | QIAGEN | Cat#74204 |
| RNeasy Lipid Tissue mini kit | QIAGEN | Cat#74804 |
| High-Capacity RNA-to-cDNA kit | Applied Biosystems | Cat#4387406 |
| iTaq Universal SYBR Green Supermix | Bio-Rad | Cat#1725121 |
| Mouse Adiponectin ELISA kit | Merck-Millipore | RRID: AB_2651034 |
| Mouse Leptin ELISA kit | Merck-Millipore | Cat#EZML-82K |
| Mouse Insulin ELISA kit | Merck-Millipore | Cat#EZRMI-13K |
| Mouse Protein Wnt-10b ELISA kit | Cusabio | Cat# CSB-EL026130MO |
| Foxp3 / Transcription Factor Staining Buffer Set | eBioscience | Cat#00-5523-00 |
| LIVE/DEAD Fixable Aqua Dead Cell Stain Kit | Invitrogen | Cat# L34966 |
| Dead cell removal kit | MACS Miltenyi Biotec | Cat#130-090-101 |
| Mouse CD11c microbeads (N418) | MACS Miltenyi Biotec | Cat#130-052-001 |
| Mouse CD4 microbeads (L3T4) | MACS Miltenyi Biotec | Cat#130-049-201 |
| Mouse IL-10 ELISA kit | eBioscience | RRID: AB_2574996 |
| Mouse IL-6 ELISA kit | eBioscience | RRID: AB_2574986 |
| Mouse IL-12p70 ELISA kit | eBioscience | RRID: AB_2575018 |
| Mouse IL-17A ELISA kit | eBioscience | RRID: AB_2575100 |
| VECTASHIELD Mounting Medium with DAPI | VECTOR laboratories | Cat# H-1200 |
| Luminata Forte Western HRP substrate | Millipore | Cat#WBLUF0500 |
| Deposited Data | | |
| Raw data files for RNA sequencing | NCBI Gene Expression Omnibus | GEO: GSE37448 |
| Experimental Models: Organisms/Strains | | |
| Mouse: C57BL/6J | Charles River | Stock No.027 |
| Mouse: $Pparg^{tm2Rev}$ | Jackson Laboratory | RRID: IMSR_JAX:004584 |
| Mouse: $Ctnnb1^{tmKem}$ | Jackson Laboratory | RRID: IMSR_JAX:004152 |
| Mouse: $Zbtb46^{tm1Kmm}$/J | Jackson Laboratory | RRID: IMSR_JAX:018534 |
| Mouse: BALB/c | Charles River | Stock No.028 |
| Mouse: B6.Cg-Tg(TcraTcrb)425Cbn/J (OT-II) | Jackson Laboratory | RRID: IMSR_JAX:004194 |
| Oligonucleotides | | |
| See Table S1 for list of primers | N/A | N/A |
| Software and Algorithms | | |
| GSEA | Broad Institute | http://software.broadinstitute.org/gsea/index.jsp |
| IMARIS | Bitplane | http://www.bitplane.com |
| ImageJ Adiposoft | ImageJ, Galarraga et al., 2012 | http://imagej.net/Adiposoft |
| Axiovision version 4.8 | Zeiss | https://www.zeiss.com |

## CONTACT FOR REAGENT AND RESOURCE SHARING

Further information and requests for reagents may be directed to and will be fulfilled by the Lead Contact, M. Paula Longhi (m.longhi@qmul.ac.uk).

## EXPERIMENTAL MODEL AND SUBJECT DETAILS

### Mice

Male *Pparg*$^{tm2Rev}$ (Ppar$\gamma^{loxp}$), *Ctnnb1*$^{tmKem}$ (Ctnnb1$^{loxp}$), *Zbtb46*$^{tm1Kmm}$/J (Zbtb46$^{GFP}$) and B6.Cg-Tg(TcraTcrb)425Cbn/J (OT-II) mice were purchased from Jackson Laboratory (US); male C57BL/6 were purchased from Charles river laboratories (UK); male Zbtb46-Cre$^{+}$ mice were kindly provided by M. Nussenzweig (The Rockefeller University, NY). Mice were housed in temperature- and humidity-controlled rooms (22°C, 55% humidity) with a 12 h light/12 h dark cycle. Mice were fed Chow or Test Diet AIN-76A (Test Diet IPS) and given water *ad libitum*; animals were rehoused in clean cages weekly. 15 g of Z-NEST (IPS) was used as nesting material to help regulate temperature and light levels (Gaskill et al., 2012). For chimeras, 8 – 10 week old C57BL/6 male mice were γ-irradiated twice with 500 rad 3 h apart. Three hours later, mice were reconstituted by intravenous (i.v.) injection with marrow cells (3 × 10$^{6}$) that had been harvested from the femurs and tibias of conditional *Ppar*$\gamma^{-/-}$ (Ppar$\gamma^{fl/fl}$ zDC-cre$^{+}$) and control WT littermates or fetal liver cells (1x10$^{6}$) from 13 day-old *Ctnnb1*$^{-/-}$ (*Ctnnb1*$^{fl/fl}$ zDC-cre$^{+}$) or control embryos. Mice were maintained on acidified water during the critical 4-week reconstitution period. All animal work was carried out in accordance with UK government Home Office licensing procedures. Mice fed Western diet were excluded from experiments if they failed to gain more than 20% body weight.

## METHOD DETAILS

### Real-Time PCR and Expression Profiling

Total RNA was isolated from 10$^{6}$ cells using Trizol (Life Technologies) and with RNeasy MinElute cleanup kits (QIAGEN). RNA from 100 mg visceral adipose tissue was isolated using RNeasy Lipid Tissue Mini Kits (QIAGEN) following the manufacturer's instructions. RNA was quantified with Spectrostar omega (BMG Labtech). Reverse transcription to cDNA was performed using High-Capacity RNA-to-cDNA Kits (Applied Biosystems) and stored at - 80°C. Primer sequences can be found in Table S1, purchased from Invitrogen, Paisley, UK. Gene expression was performed using SYBR Green Supermix (Bio-Rad), according to the manufacturer's instructions, and analyzed using a CFX connect light cycler (Bio-Rad). Gene-relative expression was calculated using the ΔΔCT method and normalized to a reference control (GAPDH) with control sample set as 1. As ΔΔCT is not normally distributed is the geometric mean is a more appropriate representation of the data than the more commonly used arithmetic mean. To note, the propagated error is the product of multiple additive errors, thus it will usually be higher than the error implied by taking the standard error of the ΔΔCT values for each replicate.

Expression profiling was performed using publicly available raw data (ImmGen GEO: GSE37448) in R (R Core Team, 2016). Between-array RMA quantile normalization was performed and unmapped or multi-mapping probes removed prior to differential expression analysis with limma (Ritchie et al., 2015). Fold change rankings were used in GSEA to identify differentially regulated pathways (FDR < 0.25). Processed data are available on ImmGen's data browsers (http://www.immgen.org/).

### *Ex Vivo* Confocal Microscopy

Zbtb46-GFP mice were injected i.p. with fluorescently conjugated primary antibodies against LYVE-1 (eBioscience) and PECAM-1 (eBioscience) 4 hours prior to surgery. Mice were sacrificed and the mesenteric organs including the associated VAT was exteriorized and fixed in 4% PFA for 10min. Tissue was then mounted on a home-built perplex stage and viewed using a Leica SP8 confocal microscope as previously described (Arokiasamy et al., 2017). Acquired confocal images were analyzed using the 3D imaging analysis software IMARIS (bitplane, Switzerland).

### Metabolic Assays

Adiponectin and Leptin levels were measured in the serum from fasted lean and obese mice by ELISA (Merck Millipore), as well as by RT-PCR from visceral adipose tissue as described above. Similarly, fasted insulin levels were detected by ELISA (Merck Millipore). Wnt10b was detected from visceral adipose tissue homogenates by ELISA (Cusabio). For Glucose (GTT) and Insulin (ITT) tolerance tests fasted blood glucose levels were measured from initial tail bleeds at room temperature using blood glucose meter and test strips (FreeStyle Optium Neo, Abbott). Mice were administered with 1.5 mg D- Glucose/ g of body weight (Sigma) or 0.5 U Insulin/ kg of body weight (Actrapid) via intraperitoneal (i.p.) injection and blood glucose measurements were taken from the tail bleeds at 15, 30, 60, 90, 120, 180 mins after injection at room temperature.

To assess the insulin signaling, mice were injected i.v. with 5 U Insulin/ kg of body weight at room temperature. After 15 minutes the VAT and liver were harvested and tissue levels of phospho-(Ser473) Akt and total AKT were measured by western blot.

Body weight and food intake was recorded weekly. Weight of total visceral adipose tissue harvested was recorded for cell number calculations and to determine the percentage of body weight. Visceral adipose tissue was fixed in 4% PFA and embedded in paraffin. Sectioned slides were stained with hematoxylin and eosin by BCI Pathology core services (QMUL). Adipocytes size were calculated using ImageJ Adiposoft software (Galarraga et al., 2012) by researcher blinded to the tissue genotype. Three fields of view from each image per mouse were analyzed.

### Flow Cytometry Staining

Immune cells from spleen, mesenteric lymph nodes and inguinal lymph nodes were isolated after digestion with Collagenase D (Roche). For visceral adipose tissue immune cells, tissue was digested with Collagenase II (Sigma) and DNase (Sigma) for 30 min. Immune cells present in the vascular fraction were obtained after centrifugation and lysed for Red blood cells before staining. All

samples were stained with fixable Aqua Dead cell stain (Invitrogen) to exclude dead cells from analysis. Cells were stained for surface markers using the following antibodies; CD16/32-FITC, F4/80-PerCP, CD206-BV421, CD11c-BV605, CD103-APC, MHCII-AF700, CD11b-AF780, MertK-PE, CD45-PE-CF594, CD64-PE-Cy7, CD8-FITC, Ly6G-BV421, NK1.1-BV605, CD3-AF700, SiglecF-PE-CF594, CD4-PE-Cy7, B220-PerCP, CD36-APC, CD86-BV605, CD80-PE (eBioscience/ BioLegend/ BD Bioscience/ R&D Systems). Samples were stained at 4°C for 30 min and fixed at 4°C for 30 min with 1% PFA. For intracellular staining of FOXP3, samples were incubated in permeabilization/fixation buffer (eBioscience) at 4°C for 30 min and washed in permeabilization buffer before staining with antibody FOXP3-PE (Biolegend) at 4°C for 30 min in permeabilization buffer. Samples were the analyzed by flow cytometry using a LSR Fortessa (BD Biosciences) and FlowJo version 10 software.

### DC Innate Responses

Dendritic cells were isolated from the spleen and visceral adipose tissue of 3-5 pooled Zbtb46-GFP mice, by first magnetic depletion of dead cells (MACS Miltenyi Biotec) followed by $CD11c^+$ bead positive selection (MACS Miltenyi Biotec). $CD11c^+$ $MHCII^+$ $GFP^+$ $CD103^+$ CD1 or $CD11c^+$ $MHCII^+$ $GFP^+$ $CD11b^+$ CD2 were purified by cell sorting (FACSAria; BD Biosciences). Cells were plated at $10^6$ cells/well and stimulated with the TLR4 agonist GLA (IDRI) in the presence or absence of Rosiglitazone 2.5 μM (Sigma) or SB216763 20 μM (Abcam Biochemicals). In specific experiments cells were additionally stimulated with 100 ng/mL of recombinant Wnt10b (R&D Sytems). After culturing overnight, supernatant was collected and levels of IL-10, IL-6, IL-12p70 were determined by murine ELISA (eBioscience).

### DC Antigen Presentation

For DC antigen presentation, *in vivo*, chimera mice were injected intravenously with ovalbumin-specific OT-II cells purified from spleen and lymph nodes by $CD4^+$ bead positive selection (MACS Miltenyi Biotec) and labeled with CFSE 3 μM (Sigma). The next day, mice were immunized with 200 μg of Ovalbumin (Sigma) alone or together with 1 μg of LPS (Sigma). Two (after LPS) or three (after OVA alone) days later, immune cells were isolated from spleen, lymph nodes and visceral adipose tissue and stained, as described above, for analysis by flow cytometry. The dilution of CFSE in the T cell population was used as an indicator of cell division.

To evaluate OVA uptake by visceral adipose tissue phagocytic cells, 10 μg of OVA-AlexaFluor-555 (Invitrogen) was injected i.p. One hour later, visceral adipose tissue was harvested and fixed overnight in 4% PFA containing 30% Sucrose (Sigma) and stain with 0.5 μg/ml of BODIPY as per manufacturer's instructions (Invitrogen). Tissue was laid onto poly-L-lysine slides with mounting medium containing DAPI (Invitrogen). Tissue was visualized using a Zeiss Z1 fluorescence microscope (Carl Zeiss, Cambridge, UK) equipped with an AxioCam MRm Cooled monochrome digital camera and an Apotome 2 Imaging unit. Images were acquired using a Plan Apochromat 40x or 20x/0.8 NA objective and Axiovision software version 4.8.

To test allostimulatory capacity *in vitro*, spleen and visceral adipose tissue DCs were isolated, as described above, from lean and obese Zbtb46-GFP or chimera mice. $CD4^+$ T cells were harvested from the spleen of BALB/c mice by $CD4^+$ bead positive selection and labeled with CFSE 3 μM. Cells were mixed at a ratio of 1:5 ($5 \times 10^4$ $CD11c^+$ dendritic cells: $2.5 \times 10^5$ $CD4^+$ T cells / well) and incubated for 3 - 5 days, after which cells were harvested and CFSE dilution was assessed by flow cytometry and cytokine production in supernatant by ELISA.

### Western Blotting

Protein lysates were prepared from tissue or purified cDCs using RIPA buffer. Proteins were separated with SDS-PAGE and transferred to Immobilon PVDF membrane (Millipore). Membranes were blocked for 1 h at room temperature in PBST containing 5% (w/v) milk, incubated overnight at 4°C with primary antibodies and subsequently with HRP-conjugated secondary antibody (Amersham Bioscience). Antibodies against non-phospho (active) β-catenin, PPARγ, phospho-(Ser473) Akt and total Akt were purchased from Cell Signaling Technologies; antibodies against Tubulin, GAPDH were purchased from Genetex. Blotted proteins were detected using Luminata Forte Western HRP substrate (Millipore) and exposed on to Hyperfilm photo film (Amersham).

## QUANTIFICATION AND STATISTICAL ANALYSIS

Data reported in the figures represent the average of at least three independent experiments. Statistical significance was determined by Student's t test with two-tailed *P value*s of 0.05 or less. For GTT and ITT, statistical significance was evaluated with 2-way ANOVA followed by Bonferroni post-test. Data were analyzed and charts were generated using Prism 5 (GraphPad Software). Normal distribution was assessed with Prism 5 using the Kolmogorov-Smirnov and the Shapiro-Wilk normality test. Differences were considered significant at *$p < 0.05$, **$p < 0.005$, ***$p < 0.0005$, ****$p < 0.00005$; n.s. non significant.

## DATA AND SOFTWARE AVAILABILITY

### Data Resources

Raw data files for the RNA sequencing analysis have been deposited in the NCBI Gene Expression Omnibus under accession number GEO: GSE37448.

Cell Metabolism, Volume *27*

## Supplemental Information

# Visceral Adipose Tissue Immune Homeostasis Is Regulated by the Crosstalk between Adipocytes and Dendritic Cell Subsets

**Claire E. Macdougall, Elizabeth G. Wood, Jakob Loschko, Valeria Scagliotti, Féaron C. Cassidy, Mark E. Robinson, Niklas Feldhahn, Leandro Castellano, Mathieu-Benoit Voisin, Federica Marelli-Berg, Carles Gaston-Massuet, Marika Charalambous, and M. Paula Longhi**

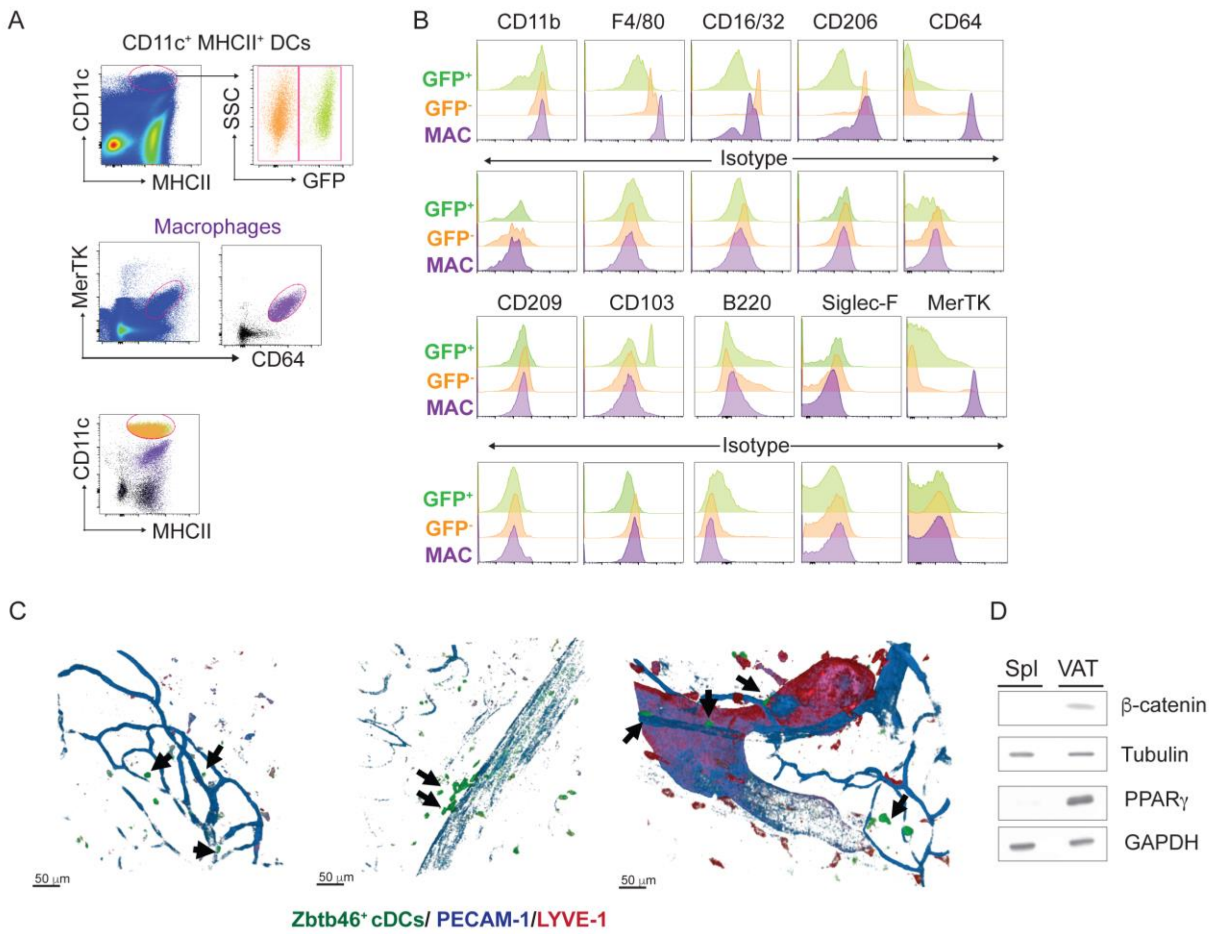


**Supplementary Figure 1. Phenotypic characterization of cDCs in VAT. Related to Figure 1.** VAT was isolated from Zbtb46-GFP⁺ mice. (A) cDCs were identified as MHCII⁺ CD11c⁺ GFP⁺ cells and macrophages as MerTK⁺ CD64⁺ cells. (B) Phenotypic characterization of MHCII⁺ CD11c⁺ GFP⁺, MHCII⁺ CD11c⁺ GFP⁻ cells and macrophages. Dot plots and histograms are representative of 3 independent experiments. (C) Mesenteric VAT and vessels from Zbtb46-GFP⁺ mice were imaged by *ex vivo* confocal microscopy. Tissue was visualized viewed using a Leica SP8 confocal microscope. GFP⁺ cDCs (green) located in close proximity (black arrows) to PECAM-1⁺ capillary vessels (BV, blue) of the VAT (left picture) but also close to mesenteric afferent lymphatics (LV, middle picture) and

LYVE-1$^{+}$ initial lymphatic vessels (iLV, red) near the gut (right picture). (D) Expression of active β-catenin and PPARγ in spleen and VAT detected by Western blot representative of 3 independent experiments.

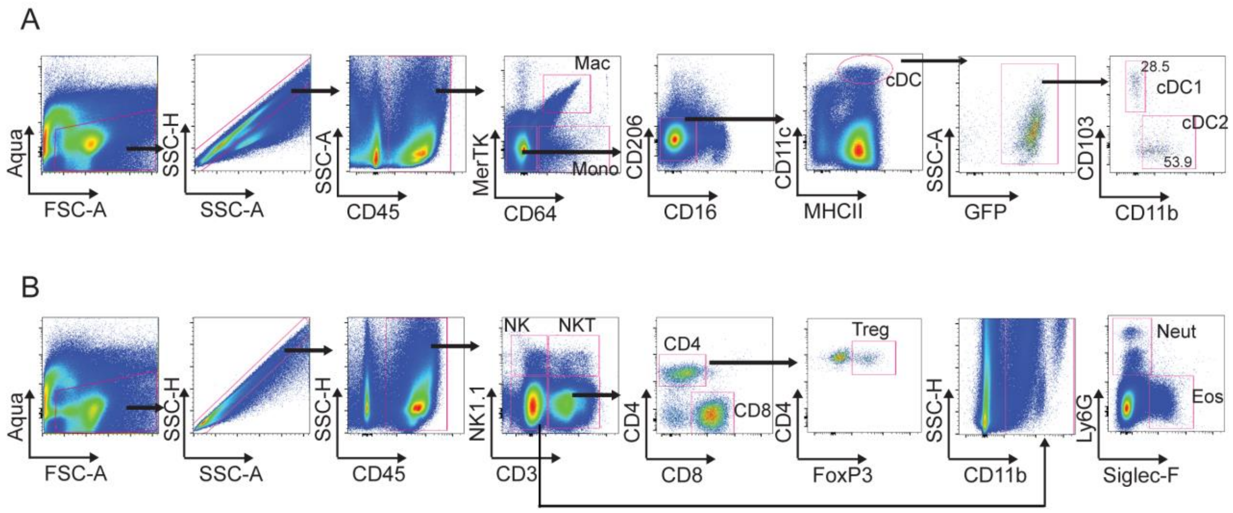


**Supplementary Figure 2. Gating strategy. Related to Figure 3 and 5.** VAT was isolated from Zbtb46-GFP$^+$ mice and immune populations were analyzed by flow cytometry. Dot plots show the gating strategy to identify cDCs, monocytes and macrophages (A, stain 1) as well as T cell populations, neutrophils, eosinophils, NK cells, NK T cells (B, stain 2).

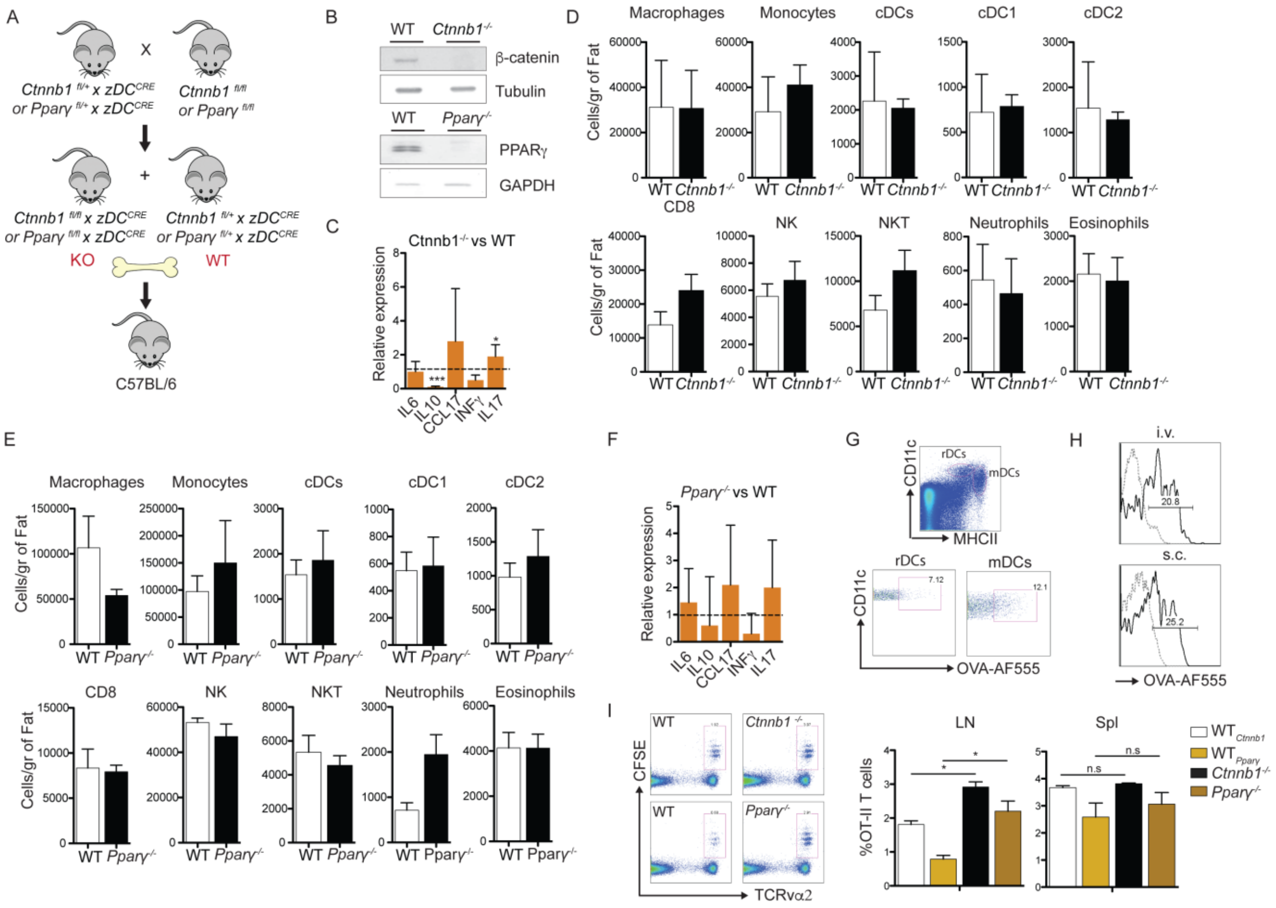


**Supplementary Figure 3. Characterization of VAT-immune infiltrates in *Ctnnb1*$^{-/-}$ and *Pparγ*$^{-/-}$ mice on chow diet. Related to Figure 3.** (A) Scheme showing experimental mice model**.** (B) Western blot showing lack of β-catenin and PPARγ expression in *Ctnnb1*$^{-/-}$ and *Pparγ*$^{-/-}$ mice respectively. (C) Quantitative RT PCR analysis for the gene expression in SVF from *Ctnnb1*$^{-/-}$ and WT mice in resting conditions. Expression levels of all genes were normalized against GAPDH RNA. Bars represent mRNA expression of conditional KO compared to WT mice, which was set at 1 as indicated with a dotted line. Error bars indicate the geometric mean of 5 biological replicates. (D-E) VAT was digested from lean *Ctnnb1*$^{-/-}$ (D), *Pparγ*$^{-/-}$ (E) and respective WT control mice. Total numbers of infiltrated immune cells identified as described in Supplementary Figure 2, were quantified by flow cytometry. Bars represent the mean ± SEM (n=6). (F) As in (C) but quantitative RT PCR

analysis for the gene expression in SVF from *Pparγ*$^{-/-}$ and WT mice in resting conditions. (G) Dot plots indicate *in vivo* AF555-OVA uptake by resident and migratory cDCs in LN. (H) Histograms indicate *in vivo* AF555-OVA uptake by VAT-cDCs after i.v. and s.c. immunization. (I) CFSE-labeled OT-II cells were transferred i.v. one day before inoculation of 200 μg OVA plus 1 μg of LPS i.p. Two days later, OT-II cell proliferation was assessed by flow cytometry. Dot plots show CFSE dilution of OT-II cells in LN (left). Bar graphs represent the average frequency of division of proliferating OT-II T cells in LN and spleen from 3 independent experiments (n=6).

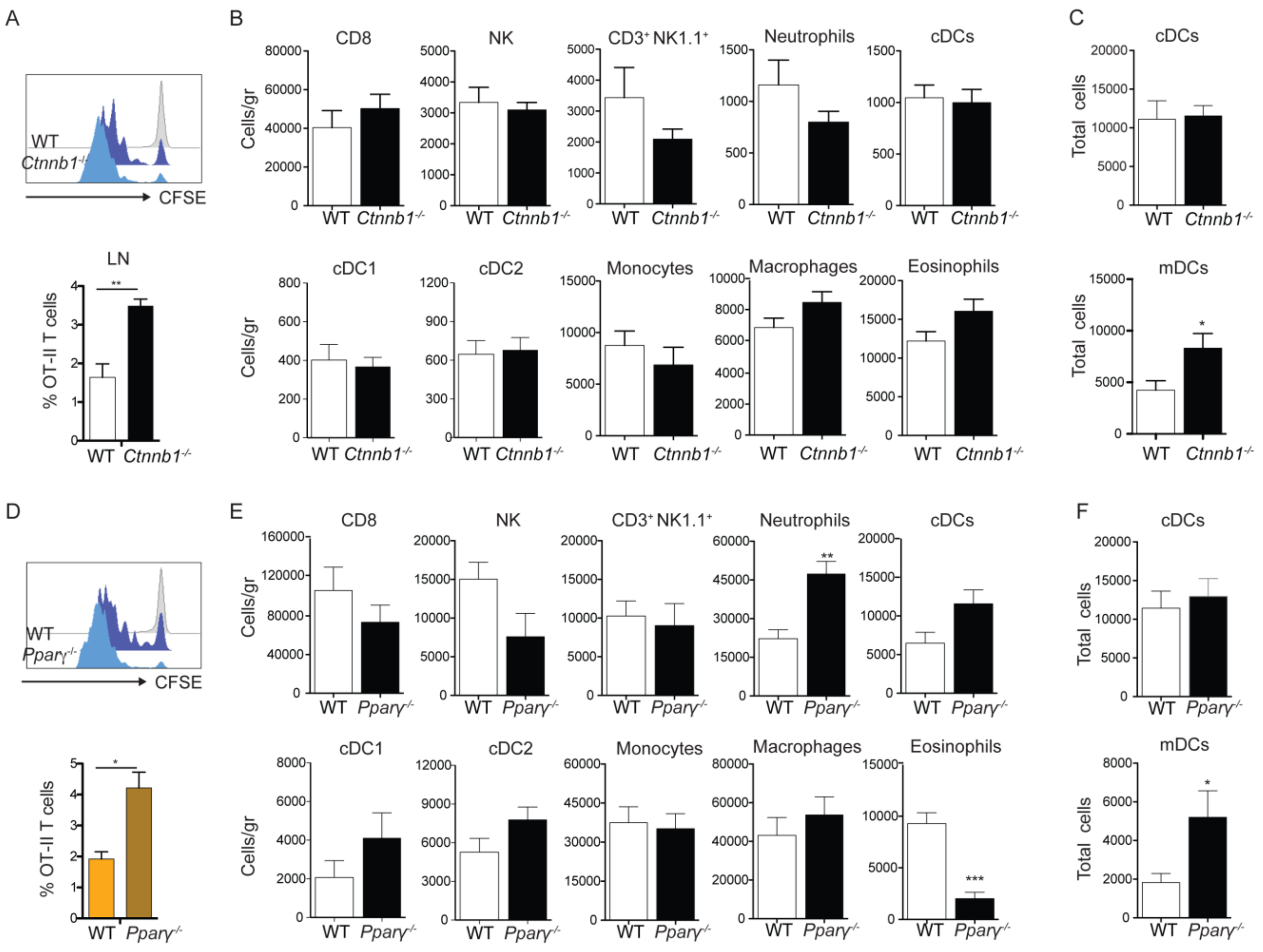


**Supplementary Figure 4. Characterization of VAT-immune infiltrates in *Ctnnb1*$^{-/-}$ and *Pparγ*$^{-/-}$ mice on WD. Related to Figure 5.** (A) CFSE-labeled OT-II cells were transferred i.v. one day before inoculation of 200 μg OVA i.p in *Ctnnb1*$^{-/-}$ and WT control mice. Three days later, OT-II cell proliferation was assessed by flow cytometry. Histogram shows CFSE dilution of OT-II cells in LN. Bar graphs represent the average frequency of division of proliferating OT-II T cells in LN in 2 independent experiments. *Ctnnb1*$^{-/-}$ (B-C), *Pparγ*$^{-/-}$ (E-F) and respective control mice were fed a WD for 12 weeks. Immune cells were identified as described in Supplementary Figure 2. Bar graphs represent the total numbers of immune cells per gram of VAT (B) and total cell numbers in LN (C) from *Ctnnb1*$^{-/-}$ and WT control mice. (D) *In vivo* OVA presentation as in (A) but in *Pparγ*$^{-/-}$ and WT control mice. (E-

F) Similar to (B-C) but immune cell numbers were quantified in *Ppar*$\gamma^{-/-}$ and WT control mice. Bars represent the mean ± SEM (n=6).

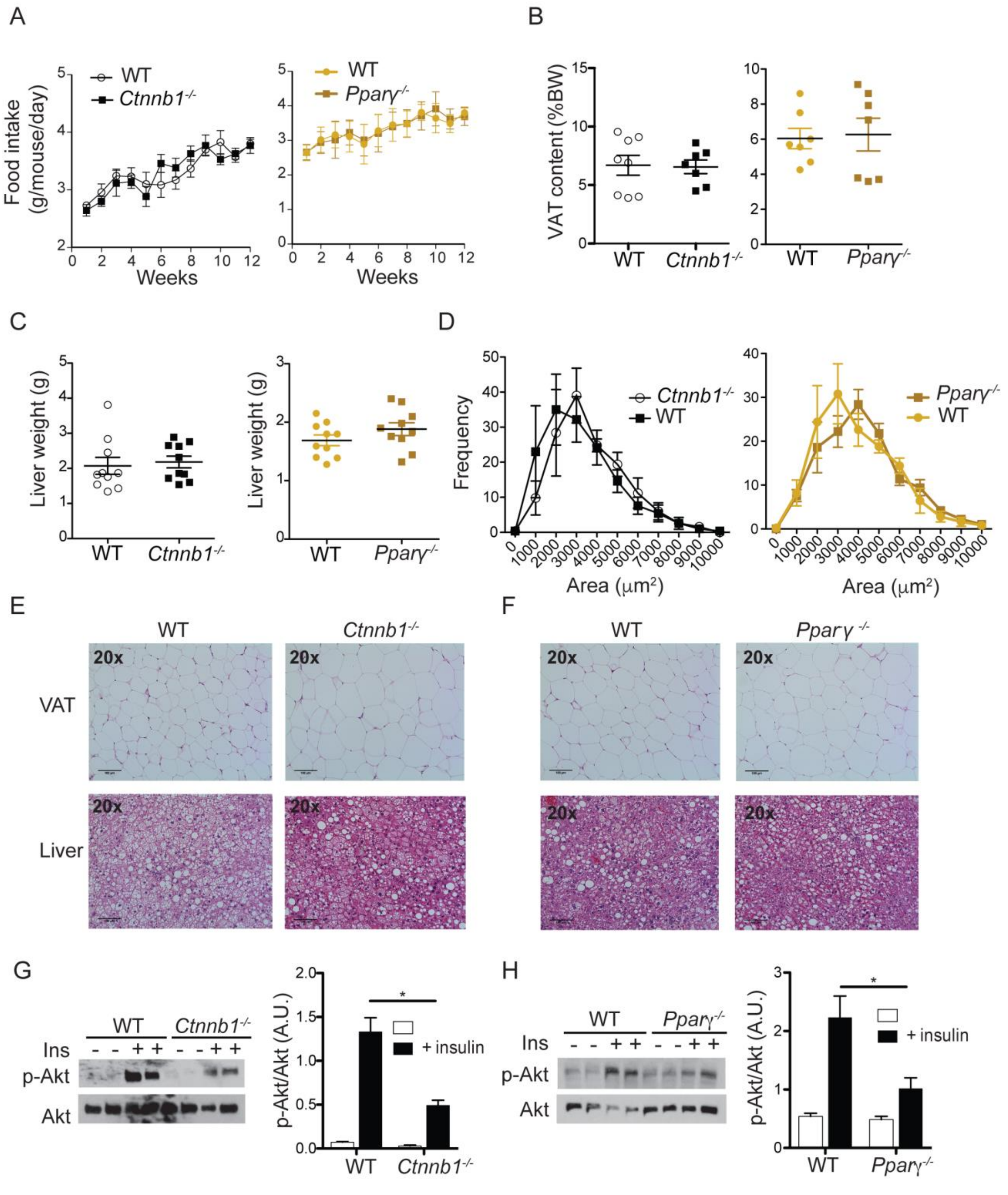


**Supplementary Figure 5. Minor changes in adipocyte function and size in *Ctnnb1$^{-/-}$* and *Pparγ$^{-/-}$* mice on WD. Related to Figure 6.** *Ctnnb1$^{-/-}$* and *Pparγ$^{-/-}$* and WT control

mice were fed a WD for 12 weeks. (A) Food intake was monitored weekly. Bars represent the mean ± SEM (n=10). (B) VAT content was calculated as percentage of body weight. Graphs represent the mean ± SEM. (C) Liver weight at 12 weeks of WD. Each dot represents an individual mouse. (D) Frequency distribution of adipocyte area isolated from VAT. Frequency of cell area is expressed in number of cells per area bin using ImageJ Adiposoft software. (E-F) Representative H&E staining from VAT and liver from *Ctnnb1*$^{-/-}$ (E) and *Pparγ*$^{-/-}$ (F) and WT control mice. (G-H) Western blot analysis of Liver extracts showing phosphor-(Ser473) Akt (p-Akt) levels under control and insulin treatment. Graphs show densitometry of p-Akt/Akt ratios in arbitrary units (A.U.).*p<0,05, **p<0,01 and ***p<0.0005.

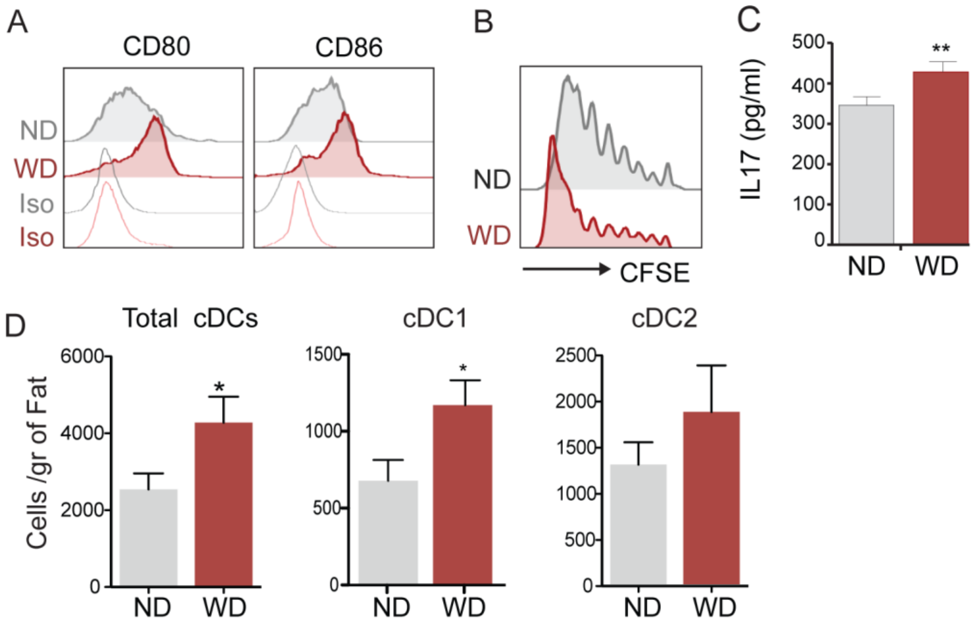


**Supplementary Figure 6. WD induces activation of VAT-cDCs. Related to Figure 7.**

Zbtb46-GFP$^{+}$ mice were fed a chow or WD diet for 12 weeks. (A) Expression of CD80 and CD86 in VAT-cDC isolated from chow or WD fed mice. Histograms are representative of 3 independent experiments. (B) VAT-cDCs were purified and plated with purified allogeneic Balb/c CD4$^{+}$ T cells (1:5) for 5 days. T cell proliferation was assessed by CFSE dilution by flow cytometry. Histograms are representative of 3 independent experiments. (C) IL-17 production by allogeneic T cells was measured in supernatant by ELISA. (D) Numbers of VAT-cDC were calculated by flow cytometry. Bars represent the mean ± SEM (n=10).

**Table S1.**
Related to STAR Methods Key Resource Table (Oligonucleotides section): List of primers used in this paper

| **Oligonucleotide name** | **Sequence (5' to 3')** |
|---|---|
| IL10_forward | AAA CAA AGG ACC AGC TGG AC |
| IL10_reverse | TTC CGA TAA GGC TTG GCA AC |
| TNFα_forward | TCG TAG CAA ACC ACC AAG TG |
| TNFα_reverse | TTT GAG ATC CAT GCC GTT GG |
| IL6_forward | TAG TCC TTC CTA CCC CAA TTT CC |
| IL6_reverse | TTG GTC CTT AGC CAC TCC TTC |
| IL1_forward | AAC CTG CTG GTG TGT GAC GTT C |
| IL1_reverse | CAG CAC GAG GCT TTT TTG TTG T |
| INFγ_forward | CAG CAA CAG CAA GGC GAA A |
| INFγ_reverse | CTG GAC CTG TGG GTT GTT GAC |
| IL17_forward | AAA GCT CAG CGT GTC CAA AC |
| IL17_reverse | TTC TGG AGC TCA CTT TTG CG |
| CCL2_forward | GCT GGA GCA TCC ACG TGT T |
| CCL2_reverse | ATC TTG CTG GTG AAT GAG TAG CA |
| CCL17_forward | GGA TGC CAT CGT GTT TCT GA |
| CCL17_reverse | GCC TTC TTC ACA TGT TTG TCT TTG |
| Apidoq_forward | GAC GTT ACT ACA ACT GAA GAG C |
| Apidoq_reverse | CAT TCT TTT CCT GAT ACT GGT C |
| Leptin_forward | TGA GTT TGT CCA AGA TGG ACC |
| Leptin_reverse | GCC ATC CAG GCT CTC TGG |
| GAPDH_forward | GGC TCA TGA CCA CAG TCC A |
| GAPDH_reverse | CAC ATT GGG GGT AGG AAC AC |
| TGFβ_forward | CCCGAAGCGGACTACTATGC |
| TGFβ_reverse | ATAGATGGCGTTGTTGCGGT |
| WNT1_ forward | ATT TTG CGC TGT GAC CTC TT |
| WNT1_reverse | AGC AAC CTC CTT TCC CAC TT |
| WNT2_forward | GGT CAG CTC TTC ATG GTG GT |
| WNT2_reverse | GGA ACT GGT GTT GGC ACT CT |
| WNT3a_forward | TCG GAG ATG GTG GTA GAG AAA |
| WNT3a_reverse | CGC AGA AGT TGG GTG AGG |
| WNT5b_forward | TCT CCG CCT CAC AAA AGT CT |
| WNT5b_reverse | CAC AGA CAC TCT CAA GCC CA |
| WNT6_forward | GCG GAG ACG ATG TGG ACT TC |
| WNT6_reverse | ATG CAC GGA TAT CTC CAC GC |
| WNT7a_forward | GAC AAA TAC AAC GAG GCC GT |
| WNT7a_reverse | GGC TGT CTT ATT GCA GGC TC |
| WNT8b_forward | CCA GAG TCC CGG GAG GTA G |
| WNT8b_reverse | GAG ATG GAG CGG AAG GTG T |
| WNT9a_forward | GGA CAA CCT CAA GTA CAG CAG |
| WNT9a_reverse | TCC ACT CCA GCC TTT ATC ACC |
| WNT9b_forward | ACA GCA CCA AGT TCC TCA GC |
| WNT9b_reverse | CTT GCA GGT TGT TCT CAG GC |
| WNT10a_forward | CCA CTC CGA CCT GGT CTA CTT TG |

| | |
|---|---|
| WNT10a_reverse | TGC TGC TCT TAT TGC ACA GGC |
| WNT10b_forward | AAT GCG GAT CCA CAA CAA CA |
| WNT10b_reverse | TTC CAT GGC ATT TGC ACT TC |
| WNT11_forward | TGC TTG ACC TGG AGA GAG GT |
| WNT11_reverse | AGC CCG TAG CTG AGG TTG T |
| Fabp4_forward | ATGTGCGACCAGTTTGTG |
| Fabp4_reverse | TTTGCCATCCCACTTCTG |
| Fabp5_forward | TGAAGGAGCTAGGAGTGGGAA |
| Fabp5_reverse | TGCACCATCTGTAAAGTTGCAG |
| Dgat1_forward | GGCCTTCTTCCACGAGTACC |
| Dgat1_reverse | GGCCTCATAGTTGAGCACG |
| CD36_forward | TCTTTCCTGCAGCCCAATG |
| CD36_reverse | AGCCTCTGTTCCAACTGATAGTGA |
